# Molecular characterization and cell type composition deconvolution of fibrosis in NAFLD

**DOI:** 10.1038/s41598-021-96966-5

**Published:** 2021-09-10

**Authors:** Lorena Pantano, George Agyapong, Yang Shen, Zhu Zhuo, Francesc Fernandez-Albert, Werner Rust, Dagmar Knebel, Jon Hill, Carine M. Boustany-Kari, Julia F. Doerner, Jörg F. Rippmann, Raymond T. Chung, Shannan J. Ho Sui, Eric Simon, Kathleen E. Corey

**Affiliations:** 1 Harvard Chan Bioinformatics Core, Department of Biostatistics, Harvard T.H. Chan School of Public Health, 401 Park Dr, Boston, MA 02215 USA; 2 Liver Center, Division of Gastroenterology, Massachusetts General Hospital, 55 Fruit St, Boston, MA 02114 USA; 3 Harvard Medical School, Boston, MA USA; 4 Boehringer Ingelheim Pharma GmbH & Co. KG, Birkendorfer Str. 65, 88937 Biberach Riss, Germany; 5 Boehringer Ingelheim Pharmaceuticals, Inc., Ridgefield, CT USA

**Keywords:** Computational biology and bioinformatics, Computational models, Data integration, Data mining, Data processing, Genome informatics, Transcription, Transcriptomics, Hepatology

## Abstract

Non-alcoholic fatty liver disease (NAFLD) is the most common cause of liver disease worldwide. In adults with NAFLD, fibrosis can develop and progress to liver cirrhosis and liver failure. However, the underlying molecular mechanisms of fibrosis progression are not fully understood. Using total RNA-Seq, we investigated the molecular mechanisms of NAFLD and fibrosis. We sequenced liver tissue from 143 adults across the full spectrum of fibrosis stage including those with stage 4 fibrosis (cirrhosis). We identified gene expression clusters that strongly correlate with fibrosis stage including four genes that have been found consistently across previously published transcriptomic studies on NASH i.e. *COL1A2*, *EFEMP2*, *FBLN5* and *THBS2*. Using cell type deconvolution, we estimated the loss of hepatocytes versus gain of hepatic stellate cells, macrophages and cholangiocytes with advancing fibrosis stage. Hepatocyte-specific functional analysis indicated increase of pro-apoptotic pathways and markers of bipotent hepatocyte/cholangiocyte precursors. Regression modelling was used to derive predictors of fibrosis stage. This study elucidated molecular and cell composition changes associated with increasing fibrosis stage in NAFLD and defined informative gene signatures for the disease.

## Introduction

Non-alcoholic fatty liver disease (NAFLD), or its more severe form, non-alcoholic steatohepatitis (NASH), is a leading cause of chronic liver disease and liver-related complications worldwide^1^. However, to date, no agency-approved treatments exist, and therapeutic trials have been challenging, partly because histologic classifications from liver biopsies, the gold standard, cannot comprehensively predict disease progression and clinical outcomes in heterogeneous patient populations^2,3^. Thus, there is an unmet need to understand the underlying molecular mechanisms of fibrosis in NAFLD and define reliable biomarkers to complement traditional histologic classifications and inform therapeutic discovery.

Transcriptomics of bulk tissue samples is a powerful tool for investigating thousands of features of a single tissue sample concurrently. Consequently, transcriptomics of liver biopsies from cohorts of human NAFLD patients have revealed molecular profiles that associate with disease progression^4,5^. Yet, these studies are based on microarray technology, which has been replaced by RNA-Seq as the state-of-the art method for transcriptional profiling^6^. Furthermore, these studies and the few existing RNA-Seq studies^7–9^ are limited by small sample sizes which skew toward less advanced fibrosis stages and therefore may not fully represent the hepatic transcriptome and the complex intercellular molecular dynamics across the full spectrum of NAFLD-related fibrogenesis. The most comprehensive RNA-Seq study in this regard has just been published very recently^10^.

Recent advances in single-cell sequencing (scRNA-Seq) can provide cell type-specific molecular profiles that contribute to disease progression^11^. However, their required cell dissociation protocols and analysis can be technically laborious and costly, making it difficult to scale this process to large patient cohorts. Few studies have jointly considered bulk and single cell transcriptome profiles from liver samples to examine the complex molecular cellular dynamics that define disease severity in human NAFLD^12,13^. Computational methods can now integrate smaller single cell transcriptome studies as references to de-convolute cell type composition and cell type-specific biological profiles of bulk transcriptomic data^14,15^. This approach can be reliably scaled to investigate the dynamics of cellular composition and cell type-specific gene expression across multiple disease stages and large patient cohorts^14^.

To contribute and extend these developments, we hypothesized that the hepatic transcriptome harbors disease-defining gene signatures that can classify fibrosis severity, and that cell type-specific molecular profiles can be derived from the bulk transcriptome by computational deconvolution. We probed the hepatic transcriptomes from a cohort with liver histology across the full spectrum of fibrosis in NAFLD to identify disease-classifying gene profiles and defined candidate gene signatures. We integrated these profiles with publicly available single-cell transcriptomic data to characterize changes in cell composition associated with fibrosis severity and evaluated the contribution of major cell types within the candidate gene signatures. We identified gene signatures and validated them with an independent NAFLD dataset of comparable histologic spectrum. This study provides comprehensive insights into molecular, cellular, and functional profiles of fibrosis in NAFLD.

## Results

### Clinical and histopathologic characteristics

Table 1 summarizes the clinical characteristics of the study cohort (n = 143). Mean patient age (years ± SD) ranged from 43.7 ± 11.4 in those with normal histology (n = 31) to 60.8 ± 5.9 in those with stage 4 fibrosis. (F4 = 12). Women composed the majority of the cohort, ranging from 90.3% of those with normal liver histology to 50% of those with NAFLD fibrosis stage 3. The mean body mass index (BMI) in the cohort ranged from 36.7 to 47.1 kg/m^2^.

**Table 1:** Characteristics of the patient cohort.

Liver histology	Normal histology	NAFLD fibrosis stage 0	NAFLD fibrosis stage 1	NAFLD fibrosis stage 2	NAFLD fibrosis stage 3	NAFLD fibrosis stage 4
N (%)	31 (21.7)	35 (24.5)	30 (21.0)	27 (18.9)	8 (5.6)	12 (8.4)
Age, years (SD)	43.7 (11.4)	45.1 (12.7)	44.4 (14.5)	44.0 (13.0)	50.4 (9.7)	60.8 (5.9)
Sex, female—yes (%)	28 (90.3)	25 (71.4)	20 (66.7)	19 (70.4)	4 (50.0)	7 (58.3)
Site code—MGH (%)	15 (48.4)	26 (74.3)	21 (70.0)	19 (70.4)	8 (100.0)	11 (91.7)
**Biopsy type**						
Explant	0 (0.0)	0 (0.0)	0 (0.0)	0 (0.0)	0 (0.0)	8 (66.7)
Extra pass (percutaneous biopsy)	0 (0.0)	0 (0.0)	1 (3.3)	0 (0.0)	0 (0.0)	1 (8.3)
Weight loss surgery (wedge biopsy)	31 (100.0)	35 (100.0)	29 (96.7)	27 (100.0)	8 (100.0)	3 (25.0)
Diabetes mellitus—yes (%)	8 (25.8)	11 (31.4)	12 (40.0)	14 (51.9)	7 (87.5)	9 (75.0)
BMI, kg/m^2^ (SD)	44.9 (5.9)	46.4 (7.4)	44.0 (7.8)	47.1 (7.3)	42.9 (7.6)	36.7 (4.7)
ALT, U/L (SD)	23.0 (8.8)	36.4 (30.8)	40.2 (19.6)	59.1 (38.9)	53.0 (34.9)	36.8 (20.2)
AST, U/L (SD)	18.5 (8.5)	26.9 (19.6)	29.2 (13.0)	43.7 (23.7)	44.8 (27.6)	50.4 (35.5)
HDL, mg/dL (SD)	47.7 (11.9)	46.4 (12.4)	41.9 (11.3)	38.8 (10.3)	32.6 (7.2)	42.2 (18.8)
Triglycerides, mg/dL (SD)	106.5 (50.6)	137.2 (70.3)	137.2 (69.3)	180.1 (inf)	166.9 (56.7)	122.6 (35.0)
NASH, N (%)	0 (0.0)	9 (25.7)	21 (70.0)	26 (96.3)	7 (87.5)	6 (50.0)

As expected, histological scores, including steatosis grade, hepatocyte ballooning grade, lobular inflammation grade, NAFLD activity score and fibrosis stage correlated with one another. Histologic covariates are also moderately correlated with aspartate aminotransferase (AST) and alanine aminotransferase (ALT) levels and other clinical metrics (such as BMI, diabetes or triglyceride level etc., see Fig. S4a).

### Morphometric features complement disease staging and cell type composition in tissue

To complement the histopathology based grading of fibrosis, we generated continuous sample-level fibrosis scores from digital image features (ImageScore). An overview of the analysis workflow is shown in Fig. S1. The continuous fibrosis scores correlated well with the standard ordinal fibrosis scores assigned by histopathologists on biopsy (Figs. S2, S3). Fig. S2A and S2B depict the PCA score plots of the independent latent variables (tiles) used to generate the predictive model. The tiles clustered into four groups and the ratio of tiles in two of these groups associated strongly with fibrosis stage (Fig. S2C). This correlation was driven by the abundance of collagen and voids (Fig. S2D).

### Hepatic gene expression and functional profiles associate with fibrosis

**Figure 1:**
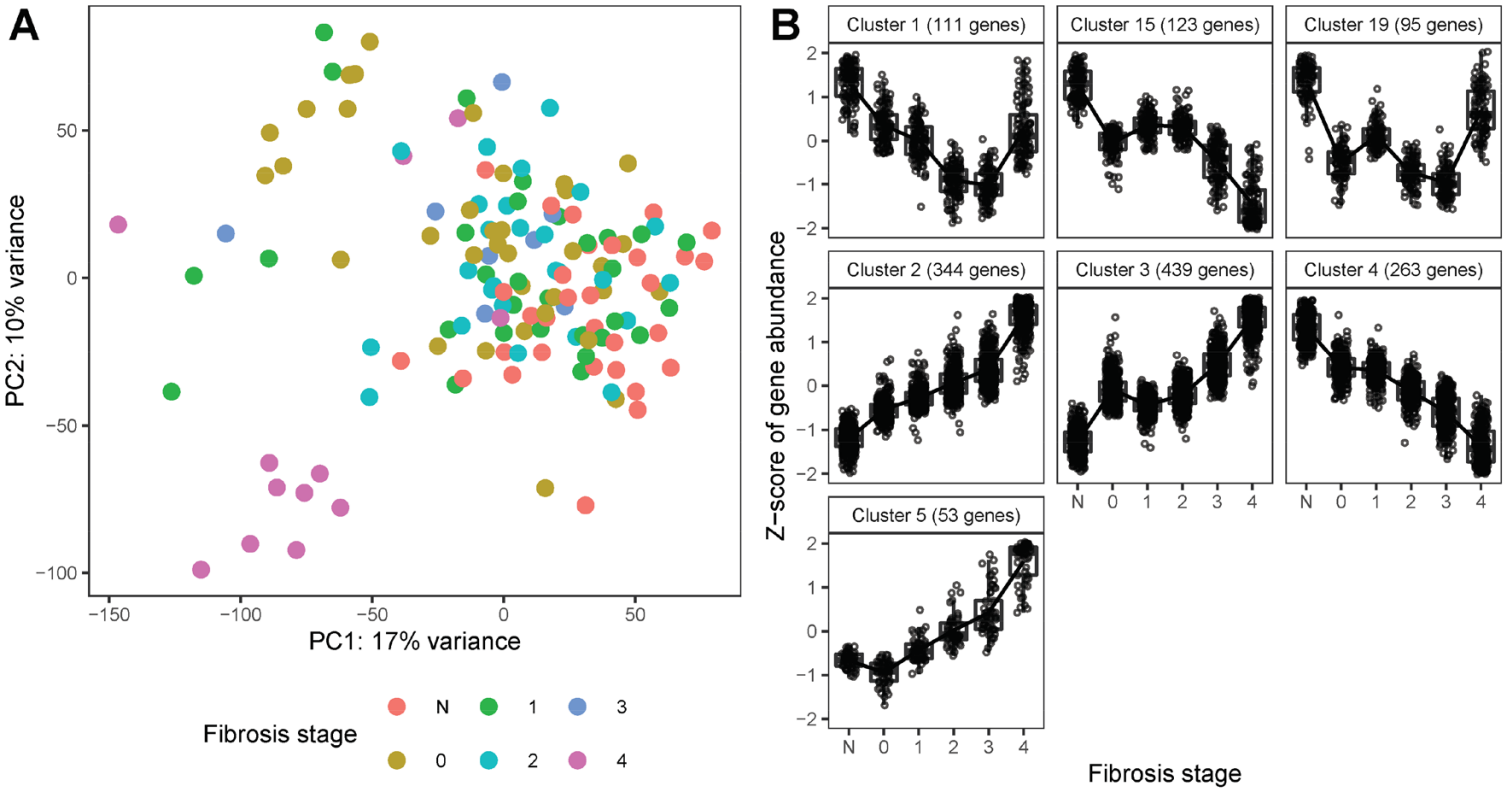
RNASeq analysis. (**A**) PCA plot of all samples. Colors represent different fibrosis stages, where N corresponds to the Normal group. (**B**) Gene expression patterns of DE genes. The Z-score represents the scaled transformation of the log2 normalized counts. Only clusters with more than 50 genes are represented.

The global data structure of the 143 samples was examined by PCA. According to Fig. 1A, the first two components of the PCA explained 17% and 10% of the observed variation in gene expression, respectively. There was a moderate clustering of samples with regard to fibrosis stage for advanced stage F3 (lower PC1) and F4 (low PC1 and low PC2). We also checked the correlation of all variables with gene expression by PCA analysis (Fig. S4B) and included the confounding variables in the DESeq2 model for differential expression (DE) analysis as described in the Methods. As noted previously, female samples were enriched in the cohort, and although sex was controlled for in the analysis, identification of DE genes in this study might be biased towards females because of the sex imbalance. Additionally, as age has a low-degree correlation with fibrosis (Kendall rank correlation coefficient 0.16, P value 0.0065), inclusion of age as one of the control variables may result in some de-regulated genes associated with fibrosis remaining undiscovered in this study.

We identified a total of 2215 differentially expressed (DE) genes by combining the results of (1) pairwise comparisons between various individual stages using fibrosis stage F0 as the reference group, and (2) LRT analysis across fibrosis stages (Fig. 1). While there were no DE genes between F1 and F0, 83 DE genes were identified between F0 and normal liver histology, 66 DE genes between F2 and F0, 65 DE genes between F3 and F0, and 882 DE genes between F4 and F0 (see also volcano plots shown in Fig. S5). LRT analysis reported 2008 DE genes, of which 1198 genes were not found in pairwise comparisons. Clustering analysis identified major gene expression patterns associated with fibrosis stage as shown in Fig. 1B.

**Figure 2:**
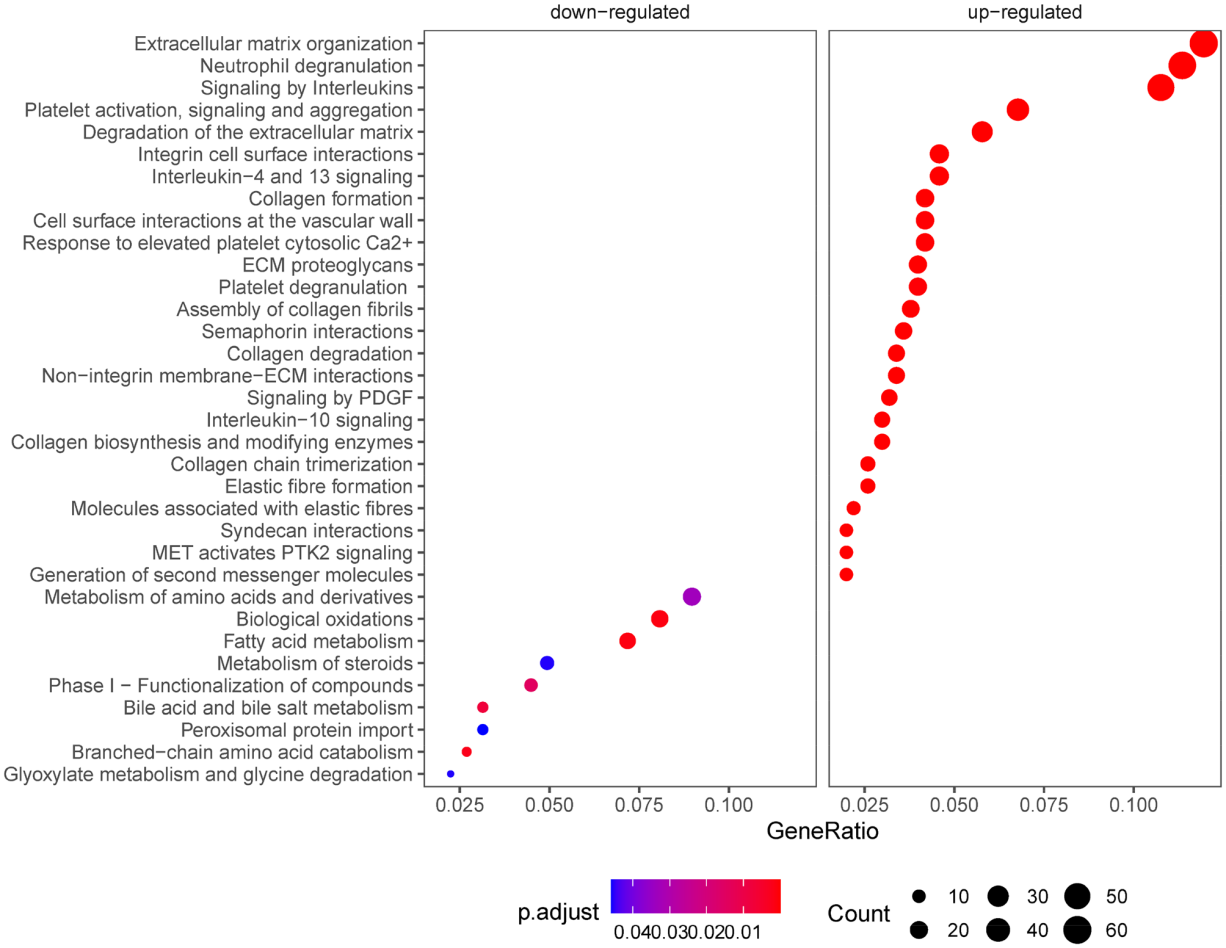
Gene set enrichment analysis. Enrichment of Reactome pathways by up-regulated (clusters 2 and 3) and down-regulated (clusters 4 and 15) DE genes. Colors represents the adjusted *P* value and the size of each dot represents the number of DE genes.

Functional analyses of the upregulated genes (clusters 2 and 3) identified pathways involved in extracellular structure organization, neutrophil degranulation, integrin signaling, interleukin signaling (IL-4, IL-13, IL-10), platelet activation and aggregation, and proteoglycan metabolism, among others (Fig. 2). In contrast, the downregulated gene profiles (clusters 4 and 15) were enriched in homeostatic hepatic functions, including catabolic and biosynthetic processes involving small molecules, organic hydroxy compounds, fatty acids and lipids, amino acids, and bile acids and salts (Fig. 2).

We further investigated and validated clusters 2 and 3 comprised of genes positively correlated with fibrosis stage (Fig. 1B) by comparing them with fibrosis associated gene lists from five previously published transcriptomic studies on NASH versus Non-NASH^5,7,8,10,16^. An overview of the identified gene sets is given in Table S1. Three of these studies have small sample sizes in advanced fibrosis stage and/or are limited to microarray technology. Accordingly, the size of the gene set that has been reported to be up-regulated with fibrosis is rather small in these three studies i.e. 86–112 genes. In contrast, the present study and the two published RNA-Seq studies with reasonable sample sizes in advanced fibrosis stage, report quite large sets of > 700 genes that are up-regulated with fibrosis in F4^10^ or positively correlated with fibrosis stage F0–F4^7^. As shown in Fig. S6, more than 50% of the genes from the larger gene sets are exclusively reported by a single study only. However, there is also a reasonable overlap of 117 genes that are reported by all three studies and almost 300 genes that are reported by at least two studies.

We also checked the overlap of all six studies listed in Table S1 and found four genes that are reported by all six studies namely *COL1A2*, *EFEMP2*, *FBLN5* and *THBS2*. All these four genes encode extracellular matrix proteins with essential functions in connective tissues as indicated by severe human phenotypes i.e. Osteogenesis imperfecta 1 (OI1) [MIM:166200] caused by mutations in *COL1A2,* Cutis laxa, autosomal recessive, 1B (ARCL1B) [MIM:614437] caused by mutations in *EFEMP2,* Cutis laxa, autosomal dominant, 2 (ADCL2) [MIM:614434] caused by mutations in *FBLN5,* and Intervertebral disc disease (IDD) [MIM:603932] which is associated with variations in *THBS2*.

### Inferring cell type composition from bulk RNASeq data

**Figure 3:**
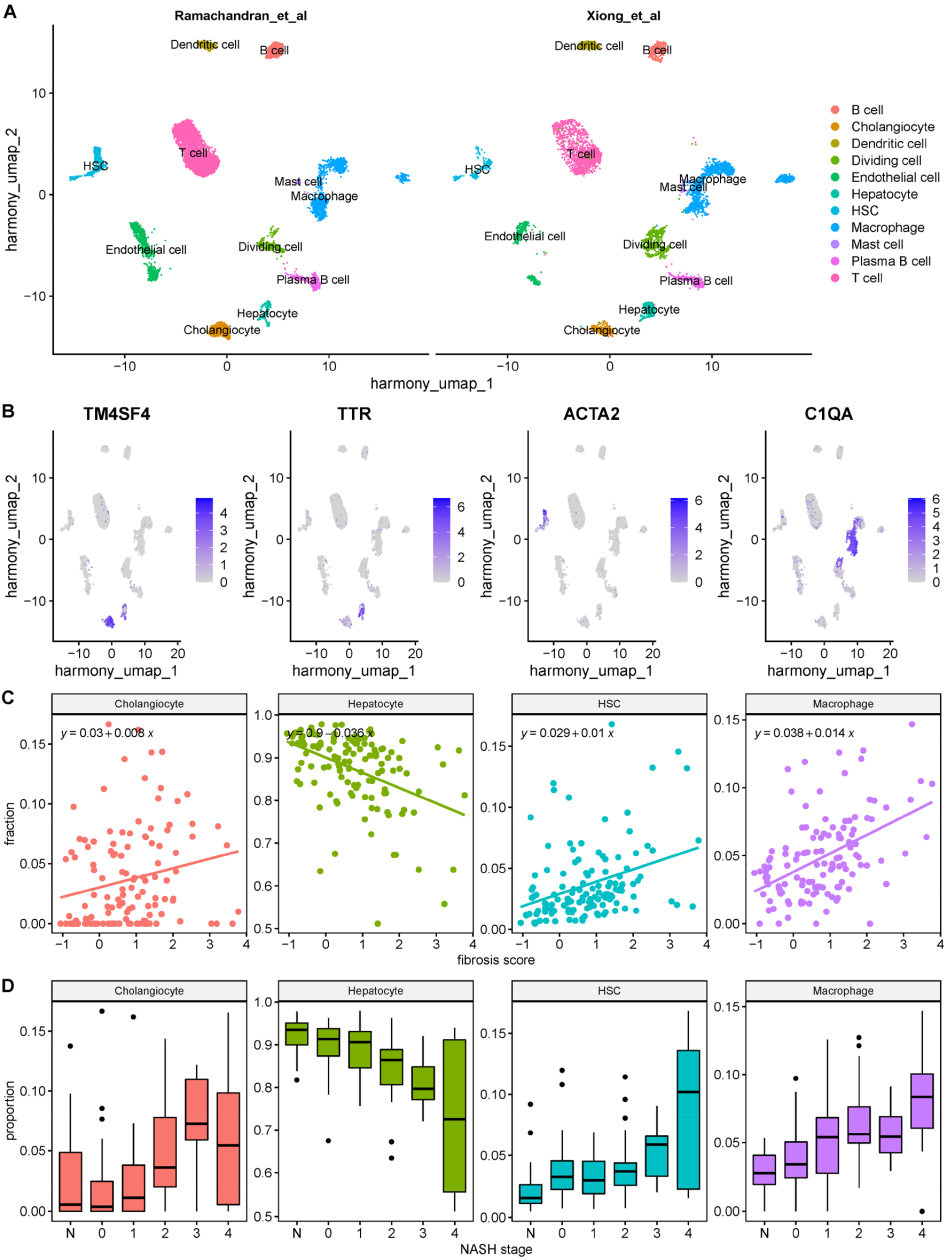
Cell composition deconvolution of the liver bulk RNA-Seq data. (**A**) Combined and integrated single cell reference data set (split UMAP view). The previously published human (11) and mouse (25) data sets have been re-analyzed, re-annotated, filtered for conserved cell types in both data sets, and finally aligned. (**B**) Validation of cell type annotation in the combined single cell reference by cell type-specific marker genes for Cholangiocytes, Hepatocytes, Hepatic Stellate Cells, and Macrophages. (**C**) Correlation between predicted cell type fraction and the continuous fibrosis score (ImageScore). (**D**) Predicted change of cell type proportions across observed NASH fibrosis stage.

We selected MuSiC^15^ for cell type deconvolution based on recommendations from comprehensive benchmarking studies^17,18^. Accordingly, MuSiC does not require a priori defined gene lists as input and is one of the preferred methods for cell type deconvolution if suitable reference scRNA-Seq datasets are available. For NASH there are two scRNA-Seq reference datasets available that cover whole liver cell populations reasonably well in healthy and disease states: One study on samples from human patients with cirrhotic livers and patients with healthy livers^12^, and one study from mice with AMLN diet-induced NASH and chow-diet controls^19^. Figure S7 illustrates the excellent performance of MuSiC in predicting cell type proportions of major liver cell types from pseudo-bulk samples which have been resampled from the two single cell reference sets (see method for details). After re-annotation and alignment of the two reference data sets, we observed good agreement of cell type clustering in both datasets (Fig. 3A). To validate the integrated reference dataset, we assessed the expression pattern of four well-known marker genes for major liver cell types. As shown in Fig. 3B, we observed consistent and cell type specific expression patterns for transmembrane 4 L six family member 4 (*TM4SF4*), transthyretin (*TTR*), actin alpha 2 smooth muscle (*ACTA2*), and complement component 1 q subcomponent A chain (*C1QA*) in cell types annotated as cholangiocytes, in hepatocytes, HSCs, and macrophages, respectively. Expression profiles of additional cell type specific markers are shown in Fig. S8A. For these four cell types, estimated changes in cell type proportions across fibrosis stage are shown in Fig. 3C, D. The largest relative variations were seen in the predicted proportions of cholangiocytes and macrophages across the fibrosis stages, with the largest proportions of these cells seen in advanced fibrosis (stage 3–4). Overall, the proportions of hepatocytes decreased, whereas proportions of cholangiocytes, HSCs, and macrophages increased with increasing fibrosis severity, as determined by both the continuous ImageScore (Fig. 3C) and the discrete fibrosis stage (Fig. 3D). Liver endothelial cells and other cell types with less than 5% predicted proportion in any fibrosis stage show a very large variability (see Fig. S8B) due to the uncertainty of the model prediction. Therefore, these cell types have not been further investigated in the present study.

### Differential expression of cell type-specific profiles in the bulk RNA-seq data

**Figure 4:**
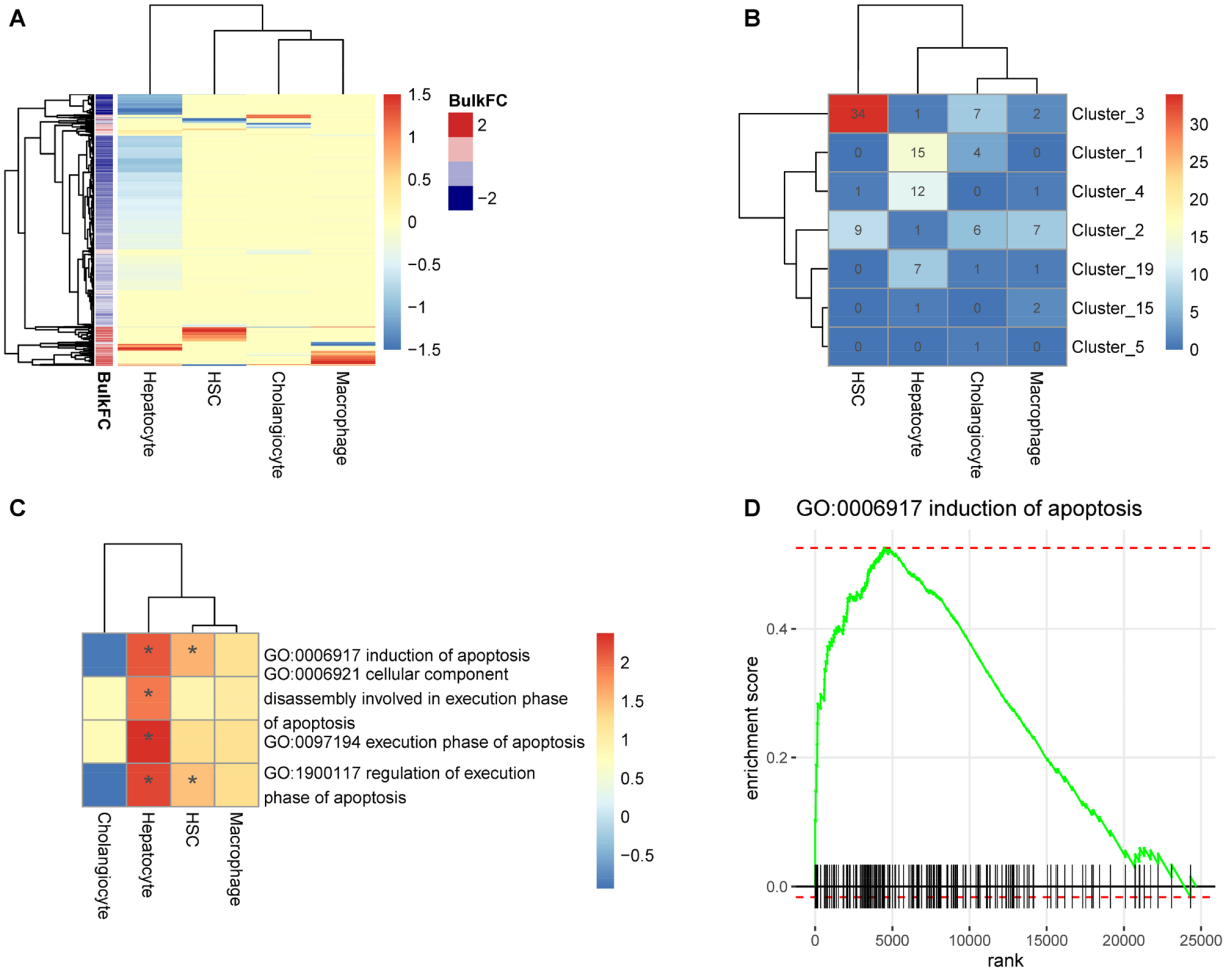
Hepatocyte-specific transcriptional up-regulation of apoptosis pathway. (**A**) Heatmap of cell-type specific differential expression, which is estimated by using regression based method with R package omicwas (see method for details), shown as log2 fold change per gene (rows) and cell type (columns) in NASH F3/F4 versus F0/Normal. (**B**) Heatmap of number of cell type-specific marker genes overlapping with disease clusters shown in Fig. 1B. (**C**) Cell type-specific functional annotation. Significantly enriched categories are marked with asterisk. (**D**) Enrichment plot of apoptosis pathway obtained from Gene Set Enrichment Analysis. Genes were ranked by the level of up-regulation (from left to right).

We determined cell type-specific differential expression patterns between advanced fibrosis (F3/F4, N = 20) and non-fibrotic NAFLD (F0, N = 66) as shown in Fig. 4. There was a dominant cluster of HSC specific up-regulated genes, with only a few down-regulated genes in F3/F4 compared to F0 (Fig. 4A). As shown in Fig. 4B, 34 of the HSC marker genes were enriched in cluster 3 from the bulk analysis (Fig. 1B) showing a positive correlation with fibrosis stage. On the other hand, the hepatocyte specific fraction was enriched in the bulk gene clusters 1 and 4 that are negatively correlated with fibrosis, except for F4 in cluster 1 (Fig. 1B). There is also a small set of genes that shows hepatocyte-specific up-regulation in F3/F4 versus normal liver histology according to the deconvolution model. Interestingly, the functional enrichment analysis indicated that this signature is enriched with pro-apoptotic genes as shown in Fig. 4C, D. This pathway is also moderately enriched in the HSC specific signature. Meanwhile, the cholangiocyte specific signal inferred by the deconvolution method showed no enrichment in the bulk gene clusters. Macrophage specific signals showed general up-regulation of genes but were not enriched in specific bulk gene clusters.

### Candidate hepatic gene signatures predict fibrosis and related biological profiles

**Figure 5:**
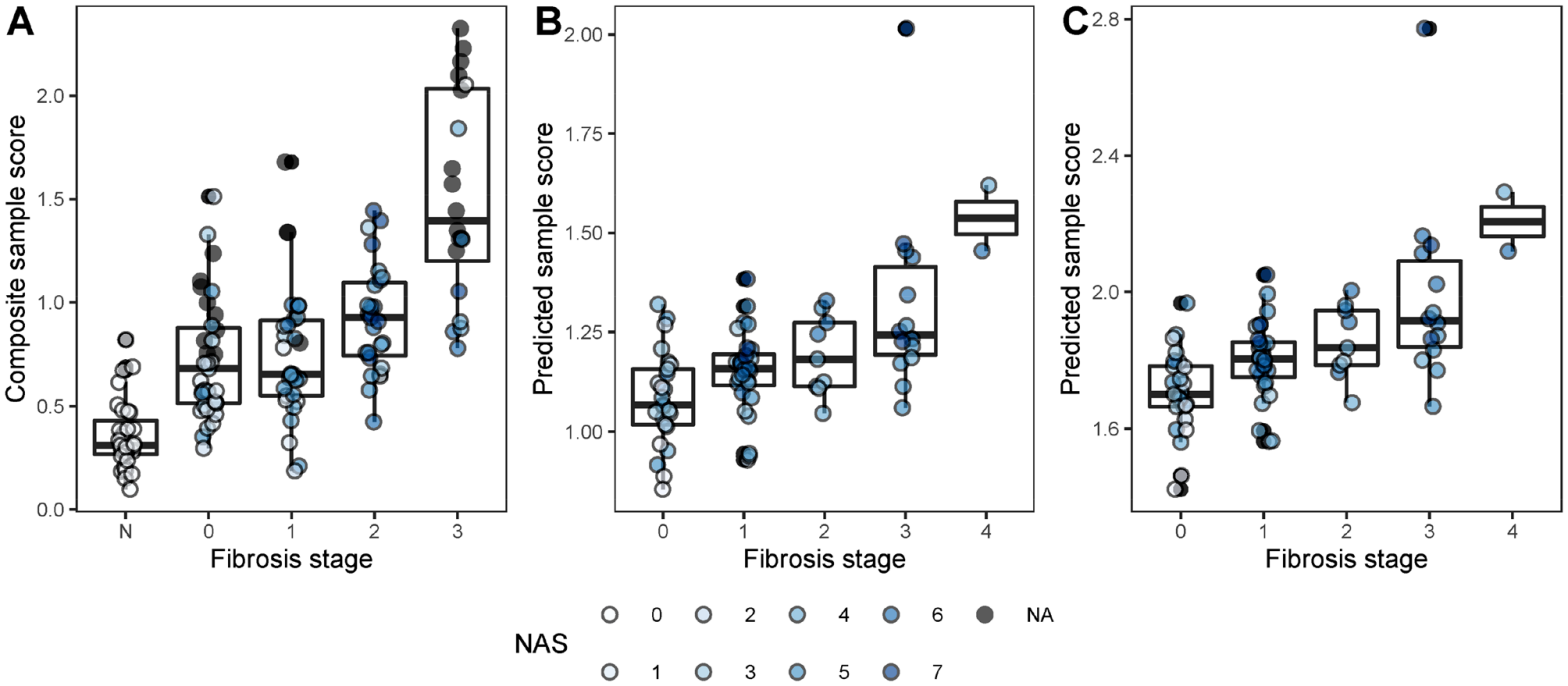
Gene signature. (**A**) Relationship between composite sample score and fibrosis stage in the NASH data. Validation of 26- (**B**) and 98-gene (**C**) signatures using data from Hoang et al. (7).

To define candidate fibrosis signatures from the bulk data, we determined that the composite sample-level gene scores from ordinal logistic modelling showed consistency with histological assessment of fibrosis severity (Kendall rank correlation coefficient of 0.57 as shown in Fig. 5A). We derived two gene signatures by selecting lambda values (the penalty parameter) that resulted in the minimum (98 genes) and a low (26 genes) mean squared error in the tenfold cross-validation of lasso regression (Fig. S9A). The two lambdas were validated using fivefold cross-validation (Fig. S9B, S9C).

Table 2 contains the two lists of signature genes. The predicted scores for fibrosis severity (referred to as signature Scores) showed high correlation with the composite sample-level gene scores (Fig. 5B, D for the 26-gene and 98-gene signatures, respectively). Additionally, we validated the two progression signatures with the data from Hoang et al.^7^, which comprised a similar spectrum of disease severity. The correlation between the signature Scores using the 26-gene signature and histological fibrosis stage was strong and further increased using the 98-gene signature. Furthermore, 20 genes from the 26-gene signature and 63 genes from the 98-gene signature belong to the up-regulated and down-regulated clusters, namely 30 genes from cluster 3, 19 genes from cluster 2 and 15 genes from cluster 4. We noted few overlapping genes including *THBS2* between our candidate signatures and previously reported signatures in NAFLD^7^, HIV associated NAFLD^22^, and hepatocellular carcinoma^23^.

**Table 2:** Candidate fibrosis signatures.

Signature	Genes
26-gene signature	AKR1B1, AL035706.1, ARL4C, ARRDC2, BTG2, COL4A1, COL4A2, CYTOR, EHD4, ERVW-1, FTOP1, GSN, HTR2A, IER5, IL27RA, INMT, LINC01725, LPAL2, NFKB2, PKM, S100A4, SOX5, TPM4, TRBC2, VIM, XYLB
98-gene signature	AC004022.2, AC007370.2, AC009974.1, AC093797.1, AC099509.1, ACOX2, ADAMTSL2, ADHFE1, AEN, AIMP1P1, AKR1B1, AL035706.1, AL121988.1, AL354890.1, AL359715.1, AL589880.1, AL591848.4, AL713866.1, APOBEC3C, ARL4C, ARRDC2, BICD2, BTG2, C2orf91, CDC42SE1, CDNF, COL4A1, COL4A2, COL5A1, CTD-2369P2.2, CXCL6, CYP51A1P2, CYTOR, DCAF6, DDI2, DTNA, EHD4, ERVW-1, F11, GLIPR2, GPNMB, GSN, H1-3, HK1, HTR2A, ICOS, IER5, IL32, INMT, IRF8, ITGAX, KPNA2, LAMC3, LCP2, LINC00939, LINC01725, LPAL2, MEAF6, MICAL1, MIR4435-2HG, NFKB2, NFYC-AS1, PGP, PIK3IP1, PKM, PLK3, PVT1, RASSF2, RGPD3, S100A11, S100A4, SERPINB9, SH2D2A, SLC16A10, SLC1A3, SLC1A7, SLC38A11, SMLR1, SOX5, STMN2, STX17-AS1, SWAP70, TAGLN2, TCEAL9, THBS2, THEMIS, THRB-IT1, TMEM51, TMSB4XP6, TNFAIP8, TOMM40L, TPM4, VIM, VOPP1, VWA7, WIPF1, XYLB, YWHAH

### Cell type and functional enrichment in the 98-gene signature

**Figure 6:**
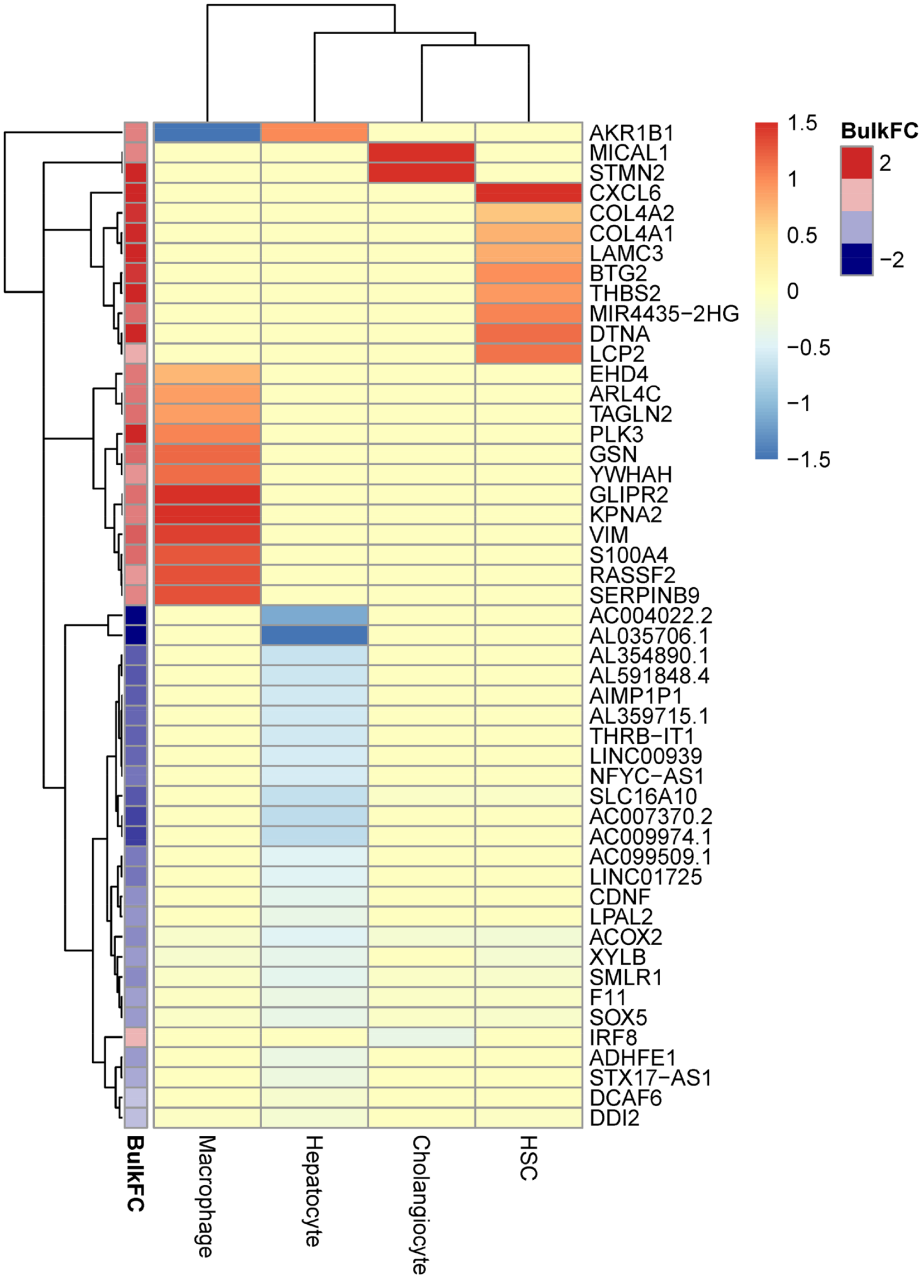
Cell type-specific differential expression of the 98-gene signature. The 98-gene signature includes 62 genes that are included in the cell type-specific marker genes with information on cell type-specific differential expression. Color code shows the cell type-specific log2 fold change in NASH F3/F4 versus Non-NASH as inferred from the deconvolution analysis. The annotation column on the right indicates the log2 fold change in the bulk RNASeq data.

Within the larger fibrosis signature, 62 genes demonstrated cell type-specific differential expression in advanced fibrosis (F3/F4), compared to non-fibrotic stages (F0). As shown in Fig. 6, two subsets of these genes showed up-regulation in macrophages or HSCs, respectively, whereas only two signature genes (*MICAL1* and *STMN2*) showed cholangiocyte-specific up-regulation. The largest subset of cell type-specific differential expression was observed in hepatocytes which comprised almost exclusively down-regulated genes. Functionally, the signature genes are involved in biological pathways annotated in focal adhesion, PI3K-AKT pathway, and PDGF signaling, among others (see Table S3).

## Discussion

Standard histologic and non-invasive NAFLD indices do not fully capture the complex biological spectrum and the heterogenous clinical outcomes in liver fibrosis. Though the underlying biological mechanisms are incompletely understood^20,21^, studies using transcriptome sequencing have provided some molecular insights in NAFLD and fibrosis^7,9,22^. Similarly, advances in single cell technologies allow for unprecedented molecular characterization of specific cell types in murine models and human samples from NAFLD^12,13,19,23^. In this study, we attempt to complement previous work (see Table S1) by including a large cohort of adults with advanced NAFLD, providing balanced representation across fibrosis stages, and leveraging relevant liver scRNA-Seq studies to identify disease-classifying molecular and cell type profiles associated with histologic fibrosis stage. We observed four extracellular matrix protein encoding genes (*COL1A2*, *EFEMP2*, *FBLN5* and *THBS2*) being up-regulated with fibrosis in NASH across all transcriptomic data sets that we have used for comparison with our data (Table S1). Interestingly, *EFEMP2* (alias *FBLN4*) and *FBLN5* are paralogous genes from the fibulin-like extracellular matrix protein family sharing 48% protein sequence identity. The fibulins protein family has five members which are characterized by the presence of EGF2-like domains and a C-terminal fibulin-type module. Fibulin-3,-4,-5 have a modified calcium binding EGF-like module at their N-terminus and are much smaller compared to fibulin-1 and fibulin-2^24^. Both, EFEMP2 and FBLN5 are essential for elastic fiber formation in connective tissues^25,26^. Proteomics studies have also shown increased fibulin-5 protein levels with hepatic fibrosis^27^ and recent functional studies show that fibulin-4 is essential for elastin and collagen fiber crosslinking and extracellular matrix assembly via lysyloxidase (LOX)^28^. *THBS2* (thrombospondin-2) also encodes a secreted ECM glycoprotein, which modestly correlates with histologic severity of NASH and fibrosis in a recent study^29^.

We deconvolved the hepatic transcriptome with a newly derived scRNA-Seq reference dataset. This computational approach showed increasing proportions of HSCs, macrophages, and transdifferentiated cholangiocytes with disease severity while hepatocyte proportion decreased in converse. Two candidate gene signatures reliably predicted fibrosis stage and reflected known and plausible biological mechanisms of disease progression. This study provides novel molecular insights into NAFLD pathogenesis and surrogates for patient stratification, prognosis, and therapeutic discovery.

The hallmark of fibrosis is an aberrant deposition of extracellular matrix (ECM) in response to hepatocyte injury through complex molecular processes, which are less understood. These fibrosis-associated molecular signals maintain profibrotic cell niches during disease progression^12,30^. In this study, the global hepatic transcriptome demonstrated molecular changes associated with fibrogenic processes in NAFLD (Fig. 1). The genes that positively correlated with increasing fibrosis stage (i.e. clusters 2 and 3) involved ECM activation and collagen processing, angiogenesis, cytoskeletal interactions, immune cell trafficking and inflammation, and platelet activation/signaling (Fig. 2). Conversely, the genes that inversely correlated with fibrosis stage (clusters 4 and 15) involved hepatocyte-specific functions such as metabolism of lipids, fatty acids, and small molecules (Fig. 2). These findings underscore important roles for immune cell trafficking^31^, platelets activation/signalling^32^, and EMC biology in fibrosis progression and point to a concomittant supression of hepatocyte function as fibrosis progressess^33^.

Cell type deconvolution with suitable scRNA-Seq reference data demonstrated that these bulk transcriptional profiles are driven in large part by changes in the proportions of liver parenchymal and non-parenchymal cell populations. The activated gene profiles were largely represented by genes associated with increasing proportions of macrophage and HSC whereas the down-regulated genes, functionally enriched with hepatocyte-specific pathways are consistent with a continous loss of hepatocyte cell proportions across fibrosis stages (Fig. 3B, C). Cell type-specific differentially expressed gene profiles were mostly observed in severe fibrosis F3/F4 compared to non-fibrotic patients F0/normal histology (Fig. 4A, B) and enriched in the candidate hepatic gene signatures as noted in Fig. 6 and Table S2.

Although the deconvolution model predicted a continous loss of hepatocytes versus other cell types with advanced fibrosis stage (Fig. 3B, C) as the major cause of the global downregulation of their metabolically-related functions (Fig. 2B), a small subset of the hepatocyte-defined genes was differentially up-regulated in severe fibrosis (Fig. 4A). This subset was functionally enriched in apoptotic pathways (Fig. 4C, D), which may partially explain the observed depletion of hepatocytes in worsening fibrosis. NAFLD results in toxic accumulation of metabolites and unhealthy organelles that drive programmed cell death in hepatocytes^34,35^. In addition to cell death, it is possible that the observed hepatocyte depletion is secondary to transdifferentiation into cholangiocytes^36^ or represents a relative reduction versus other cell types i.e. infiltrating immune cells and/or increase of hepatic stellate cells. Together, these observations are consistent with recent reports that fibrosis is also characterized by distinct niches of bipotent hepatocytes or biphenotypic progenitor cells whose fate depends on molecular cues within the diseased liver^37^.

We derived two predictive gene signatures that reliably reflected these biological profiles and correlated with histologic severity of fibrosis (Fig. 5 and Fig. S9). We focused our functional analyses on the 98 gene signature which largely included the 26 set signature as a subset (23 of 26 genes, see Table 2). Over 60% of the signature genes showed cell type-specific differential expression (Fig. 6), which underscores its inherent biological and predictive potential. The gene signatures were predictive of fibrosis stage when applied to two publicly available human NAFLD datasets^7,38^. We also compared our candidate signatures with two other published NAFLD fibrosis signatures: Only a single gene, *ADHFE1*, overlaps with the 18-gene fibrosis signature reported by^7^, while three genes overlap with the 25-genes progression signature derived by^10^ i.e. *IL32*, *STMN2*, and *DTNA*. Between the two published gene signatures there is one overlapping gene, *TNFRSF12A*. Interestingly, *IL32* has been previously reported as the top up-regulated liver transcript in NAFLD^39^. We also checked the 25-gene signature from^10^ in our cluster analysis, with 17 of the 25 genes corresponding to cluster 2 (*CCL20*, *CFAP221*, *DTNA*, *DUSP8*, *IL32*, *ITGBL1*, *STMN2*, *TNFRSF12A*), cluster #3 (*COL1A1*, *COL1A2*, *LTBP2*, *PDGFA*, *RGS4*, *THY1*) and cluster 5 (*AKR1B10*, *CLIC6*, *TYMS*) of up-regulated genes. *PDGFA* and *AKR1B10* are also the among the top three marker genes reported in this study.

Notably, the cell type resolved fibrosis signature shown in Fig. 6 underscores previously described molecular influences on the fibrotic microenvironment^12^. Molecular cues from damaged hepatocytes activate aberrant intercellular cross-talk between heterogenous monocyte-derived macrophage subpopulations^39^ and hepatic cells to orchestrate a progressive fibrotic niche^12,19,23^. Our data identified potential key drivers of these pathways within the deconvoluted macrophage, cholangiocyte, HSC, and hepatocyte specific genes in the signature. For example, the functional analysis revealed a potential role of pERK-vimentin-KPNA2 signaling genes (*VIM* and *KPNA2*) within the disease progression signature (see Table S2). This pathway was recently characterized in hepatic fibrogenesis, where VIM mediates cytoskeletal crosstalk and signal transduction through the ERK/AKT pathway to activate HSCs in fibrosis^40^. Consistent with our findings (Fig. 6), monocyte-derived macrophages express reasonably high *KPNA2* and *VIM*^41^, which suggests that infiltrating macrophages also employ this pathway and its member genes to promote fibrogenesis^19,23^. Other macrophage-annotated genes may play additional roles in hepatic cell stemness during persistent inflammatory injury (*S100A4*)^42,43^.

Moreover, the most robust functional profiles among these signatures included genes coding for ECM proteins and membrane receptors (Fig. 6 and Table S2), which were largely represented in HSCs (*CXCL6*, *COL4A2*, *COL4A1*, *LAMC3*, *BTG2*, *THBS2*) and which are also members of the overrepresented PDGF signaling pathway (see Table S2) which is known to activate epithelial-mesenchymal transition (EMT) in HSCs and promote fibrogenic signals^44^.

Together, the hepatic transcriptome revealed DE gene profiles and candidate gene signatures, which were highly enriched in pathways that plausibly reprogram HSCs, macrophages, cholangiocytes and hepatocytes toward fibrotic states in NAFLD. These proposed dynamics are not well understood and need to be further characterized.

We noted that the cell composition changes in this study do not fully reflect the heterogenous plethora of additional cell types that drive fibrosis in NAFLD, including liver sinusoidal endothelial cells (LSECs) (13), T and B lymphocytes, and other immune cells. Practically, our analysis focused on cell types that were reliably represented in the single cell reference datasets as well as the deconvoluted cell type proportions of the bulk samples. As scRNA-Seq gains momentum in hepatologic studies to generate more reference datasets, future efforts may reliably improve the sensitivity of deconvolution methods and thus resolve additional cell types and sub-populations in disease progression. Also, this will allow to replace the mouse single cell reference data by human single cell reference data once these are available for all cell types and disease conditions at reasonable coverage and resolution. However, this computational approach demonstrates dynamic cell compositions (Fig. 3), which define some of the transcriptional and functional profiles associated with fibrosis within our dataset.

Current fibrosis staging standards do not capture the full histologic continuum of liver fibrosis, particularly at the boundaries between stages (e.g., F2–F3) where cellular and phenotypic changes cannot be assessed by discrete scores. Our digital pathology model supported the deconvolution method by providing continuous morphometric scores, which reliably predicted fibrosis stage (Fig. S3) and allowed advanced statistical methods to correlate the cell type proportions with histologic stages (Fig. 3C). We acknowledge that digital pathological staging is an emerging deep learning technology, which would require larger image sample sizes beyond the scope of this study^45^.

Given the limiting challenge of acquiring clinically and demographically representative biopsy specimens for this observational study, our findings may only reflect the degree of variability and clinicopathologic classifications within this study cohort. Also, there is a risk of sampling bias due to different types of biopsies in F0–F3 (mostly derived from wedge biopsies) versus F4 (8 of 11 samples are explant). Nonetheless, compared to prior studies, the inclusion of samples from fibrosis at the most advanced stage of the disease improved histologic heterogeneity, which provides confidence that our approach has substantial potential to identify and reflect targetable pathways in NAFLD.

We are aware that the present study is descriptive and mainly based on the newly generated bulk RNASeq and histology data. Some of the observed transcriptional signals are in very good agreement with previously published data but the functional consequences of these findings remain to be clarified, as validation using orthogonal methods such as single cell RNASeq, RNA or protein in situ, and/or protein quantification assays on liver samples from appropriately-powered NAFLD patient cohorts would be required. Nonetheless, based on our data, we believe that the RNASeq method is sufficiently robust to not require additional RNA quantitation. It will be important for future studies in NASH to provide additional lines of evidence to strengthen the findings from the present study.

Herein, we characterized hepatic transcriptional and cell-composition profiles that coordinately associate with the histologic continuum of NAFLD fibrosis, to identify hepatic gene signatures that correlate with disease severity. This study provides an integrated framework to understand cellular and molecular perturbations underlying NAFLD fibrosis and inform the discovery of new biomarkers and disease therapies.

## Material and methods

### Sample collection and histologic evaluation

Subjects were selected from the Massachusetts General Hospital (MGH) NAFLD Cohort. The MGH NAFLD Cohort includes adults with suspected or established NAFLD based on imaging or liver histology. Individuals are recruited from the MGH Fatty Liver Clinic, the MGH Weight Center in Boston, MA and from the Bon Secours Health System in Richmond, VA. Subjects include adults with a standard of care liver biopsy performed at the time of bariatric surgery, adults undergoing a percutaneous liver biopsy for evaluation and staging of NAFLD and patients with NAFLD cirrhosis with liver tissue available from liver explant at the time of transplantation. Individuals in the current study were recruited between December 2010 and December 2015. Inclusion criteria were the following (1) men and women age ≥ 18 years; (2) alcohol use < 20 g daily for women or < 30 g daily for men and (3) sufficient liver tissue available for RNA sequencing. Those with other causes of chronic liver disease or those with chronic use of steatogenic medications including methotrexate, amiodarone, corticosteroids or tamoxifen were excluded.

The majority of subjects (N = 133) underwent bariatric surgery and had standard of care wedge liver biopsies performed intra-operatively, 8 subjects had NAFLD cirrhosis and underwent liver transplantation with tissue taken at the time of surgery and 2 underwent a second pass at the time of clinically indicated liver biopsy (Table 1). Half of each tissue biopsy was either immediately flash frozen or stored in RNAlater and stored at − 80 °C, while the remaining tissue was formalin-fixed and paraffin embedded for pathologic evaluation. A single hepatopathologist evaluated most biopsies (N = 117) in a blinded manner while 26 were read by clinical pathology. Normal liver histology was defined as < 5% steatosis without evidence of inflammation, hepatocyte ballooning or fibrosis. NASH was defined by the predominance of zone 3 macrovesicular steatosis, hepatocyte ballooning grade ≥ 1 with or without lobular inflammation as defined by the NASH Clinical Research Network (NASH CRN). Patients with steatosis grade > 1 (= > 5%) not meeting criteria for NASH were diagnosed with NAFL. The NASH CRN system was used to stage fibrosis on a scale from 0 (absent) to 4 (cirrhosis).

Written informed consent was obtained from each patient included in the study and the study protocol conforms to the ethical guidelines of the 1975 Declaration of Helsinki as reflected in a priori approval by the Mass General Brigham Human Research Committee.

### Morphometric image analysis

Histological liver images were broken down to tiles using Halcon Version 18 (MVTec Munich). Due to type of biopsy, the number of tiles ranged from less than 10 to more than 500 per biopsy image. For each tile, parameters to be used as features were recorded to train the predictive model, including compactness of the tissue, compactness of the voids, number of voids, equivalence radius, area of collagen, collagen area per tissue area, collagen area per section and void area. A Support Vector Regression (SVR) model was fit using the package e1071^46^. The model was trained using a 20-fold cross-validation round. Observed fibrosis scores for normal Stage (N), and F0 to F4 were set to discrete values of − 1, 0.4, respectively. Given that each sample image had a variable number of tiles, we down-sampled the number of tiles to six tiles per image. To avoid a selection bias, we repeated the down-sampling 100 times. Tiles that were not selected to train the algorithm were reserved for the validation stage. For each cross-validation round, we extracted patient-wise features by performing PCA using the tiles and taking the median of the PCA scores of the tiles that correspond to that patient. The PCA model was trained using the pcaMethods R package^47^. During this stage, a fibrosis score (designated as imageScore) for each patient was predicted using the tiles of the validation set. Therefore, after the training stage of the model, each sample had 100 different predicted imageScores. We used the median of these 100 values as the final imageScore for the assessment of fibrosis severity (see Figs. S2, S3). We used the continuous score from the morphometric image analysis to check the consistency of the pathologist-assigned fibrosis score and to assess the change in predicted cell type decomposition by deconvolution. For differential expression analysis, we used the pathologist assigned fibrosis scores.

### RNA-seq analysis

Total RNA was extracted using MagMax AM1830 kit (Fisher Scientific GmbH, Schwerte, Germany) and reverse-transcribed with 100 ng RNA using TruSeq Stranded Total RNA LT Sample Prep Kit with Ribo-ZeroTM H/M/R (Order # RS-122–2202, Illumina Inc, San Diego, CA, USA). This kit transcribes protein coding, non-coding and non-polyadenylated RNAs while cytoplasmic ribosomal RNA is depleted. The sequencing libraries were built according to manufacturer’s procedures. Sequencing was carried out at a depth of 50–55 million reads on two Illumina HiSeq systems (HiSeq 3000 for batch 1–3; HiSeq 4000 for batch 4 and 5). The Illumina TruSeq methods (cluster kit TruSeq SR Cluster Kit v3-cBot GD-410-1001, sequencing kit TruSeq SBS Kit HS- v3 50-cycle FC-410-1001) were applied as 85 bp, single reads and 8 bases index read.

The sequencing data were processed using the bcbio-nextgen RNA-Seq analysis pipeline^48^. Reads were mapped to reference genome hg19 using STAR^49^ for quality assessment and to the transcriptome using Salmon^50^ for quantification. Covariates with significant correlations with gene expression variation based on principal components analysis (PCA) (Fig. S4) were identified and controlled for further downstream analysis. Accordingly, batch, site code, age, sex, race, intergenic rate, rRNA rate, and RNA integrity number (RIN) were included in the linear model for differential expression (DE) analysis, which was restricted to protein coding genes. DE genes were identified using DESeq2^51^ in comparisons between fibrosis stage 0 and Normal liver histology, and between each fibrosis stage of 1, 2, 3, 4 and stage 0. In addition, a likelihood ratio test (LRT) was performed using the fibrosis stage as a model variable to detect genes only explained when the fibrosis stage variable was included in the model. Gene expression patterns for DE genes were computed and visualized using the DEGreport R package^52^. Functional analysis was performed in R using ReactomePA^53^, clusterProfiler^54^ for the DE genes, and g:Profiler for the signature gene set^55^, using a false discovery rate (FDR) threshold of less than 0.05 for statistical significance. Sequencing raw data is available at the GEO with accession number GSE162694.

### Cell type deconvolution of liver bulk RNASeq

Based on the performance of the cell type proportion predictions from pseudo-bulk mixtures^1^, we employed MuSiC^15,17^, which applies weighting of genes according to cross-subject and cross-cell consistency. We validated the deconvolution method and generated a combined human and mouse single cell reference data set for our approach as described in the Supplemental method section. To estimate cell type-specific differential expression based on predicted cell type proportions, we applied a regression-based method implemented in the omicwas R package^56^. We combined fibrosis stages 3 and 4 as the *disease* group denoting advanced fibrosis, and stage 0 fibrosis and normal liver histology as the *control*, non-fibrotic group. With raw expression as TPM, cell type-specific differential expression between disease and control groups was identified by the *ctassoc* function, while controlling for sex as a confounder.

### Identification of gene signatures associated with fibrosis stage

We adapted the method by Hoang et al.^7^ to define a gene signature that associates with fibrosis stage. Briefly, we modelled the relationship between the clinical classification of fibrosis stage and each gene’s expression level by fitting an ordinal logistic regression model using the variance stabilizing transformation (VST) data from DESeq2^51^. In contrast to the differential expression and functional analysis, we included also non-coding genes in this model. A weighted gene-level score was calculated based on the fitted model for each gene and each sample. Genes were ranked by the coefficient of variation of the gene-level scores, and the mean of the top 1000 genes was calculated to obtain a sample-level score indicative of fibrosis severity. Next, the composite sample-level scores were used to fit a lasso regression against gene expression. Lambda, the regularization penalty parameter was chosen to achieve a desirable number of predictor genes based on the results of k-fold cross-validation. We verified that the gene signatures were predictive of fibrosis stage using independent NAFLD RNA-Seq data sets from Hoang et al.^7^ and Fourman et al^38^ (data not shown). We also assessed the extent of enrichment of the deconvolved cell type-specific genes within the signatures.

For systematic review of previously published sets of genes that are up-regulated with fibrosis in NASH we screened the literature and gene expression repositories (GEO and ArrayExpress). We included all studies with reasonable sample size of biopsy confirmed patients with NASH and fibrosis and accessible primary data.

## Supplementary Information

Supplementary Information 1.

Supplementary Information 2.

Supplementary Information 3.

Supplementary Information 4.

## Data Availability

Raw RNASeq bulk data of the human NASH samples from the present study is available under the Gene Expression Omnibus (GEO) deposition number GSE162694. In addition, we re-processed data from the following previously published data sets: Single cell reference data set for Human liver cirrhosis (12): GSE136103. Single cell reference data set for mouse NASH model (18):(18): GSE129516. Source code to run the morphometric image analysis and cell type deconvolution can be obtained on request to the authors.

## Abbreviations

DE genes: Differentially expressed genes
ECM: Extracellular matrix
GEO: Gene Expression Omnibus
HSC: Hepatic stellate cells
LRT: Likelihood ratio test
NAFLD: Non-alcoholic fatty liver disease
NASH: Non-alcoholic steatohepatitis
PCA: Principal component analysis
scRNA-Seq: Single cell RNA-Sequencing
