# Supplementary material for: Molecular characterization and cell type composition deconvolution of fibrosis in NAFLD
Source: Sci Rep. 2021 Sep 10;11:18045. doi: 10.1038/s41598-021-96966-5 (PMC8433177; doi:10.1038/s41598-021-96966-5)
Supplement: Supplementary file 1 — Supplementary Information 1. [file 41598_2021_96966_MOESM1_ESM.docx]

**
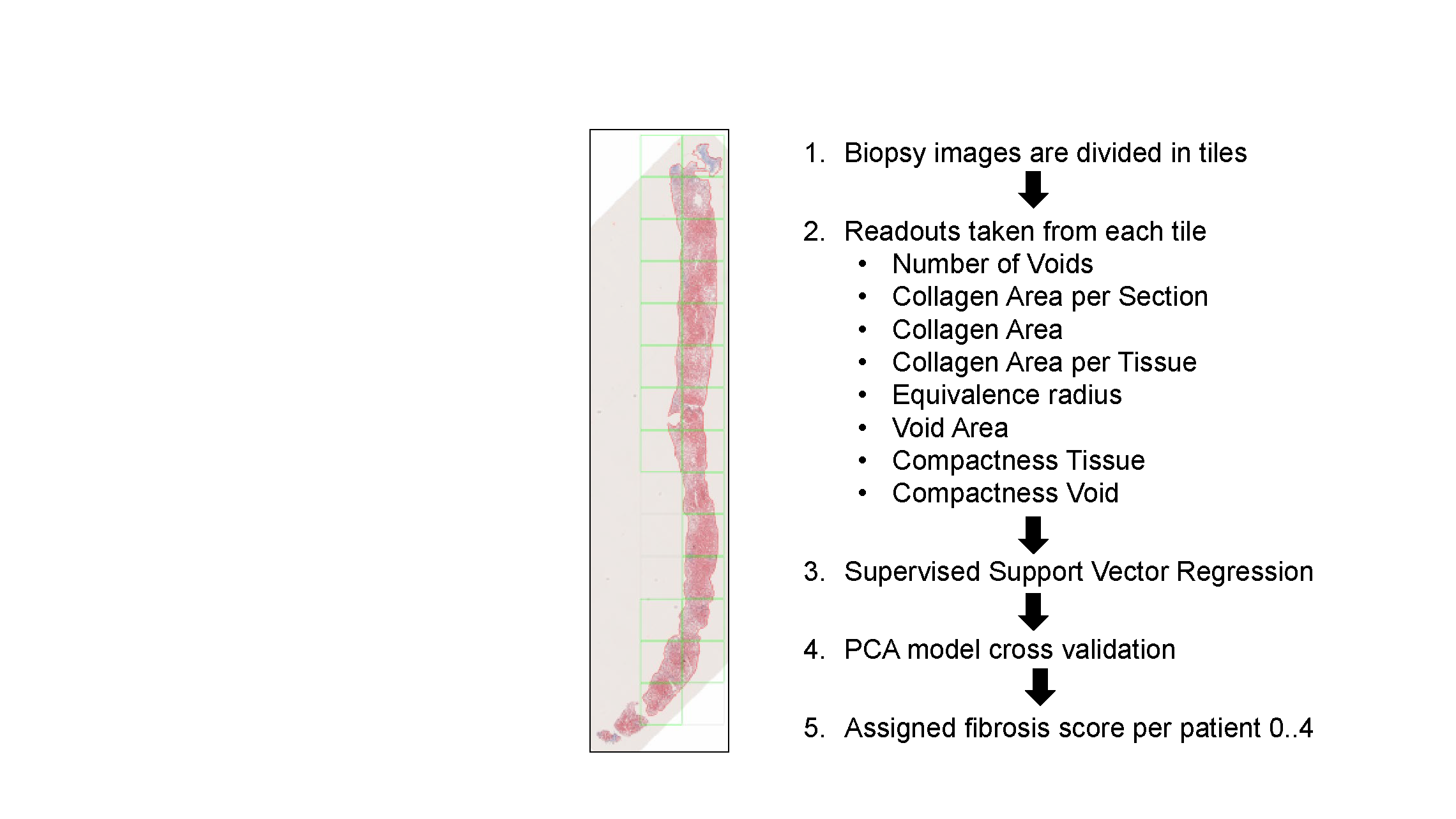
**

Fig. S1**. Workflow for morphometric image analysis.** Automated morphometric image analysis has been performed to derive continuous fibrosis scores per patients which have been used to check consistency of assigned discrete fibrosis stages and to assess change in cell type composition by deconvolution.


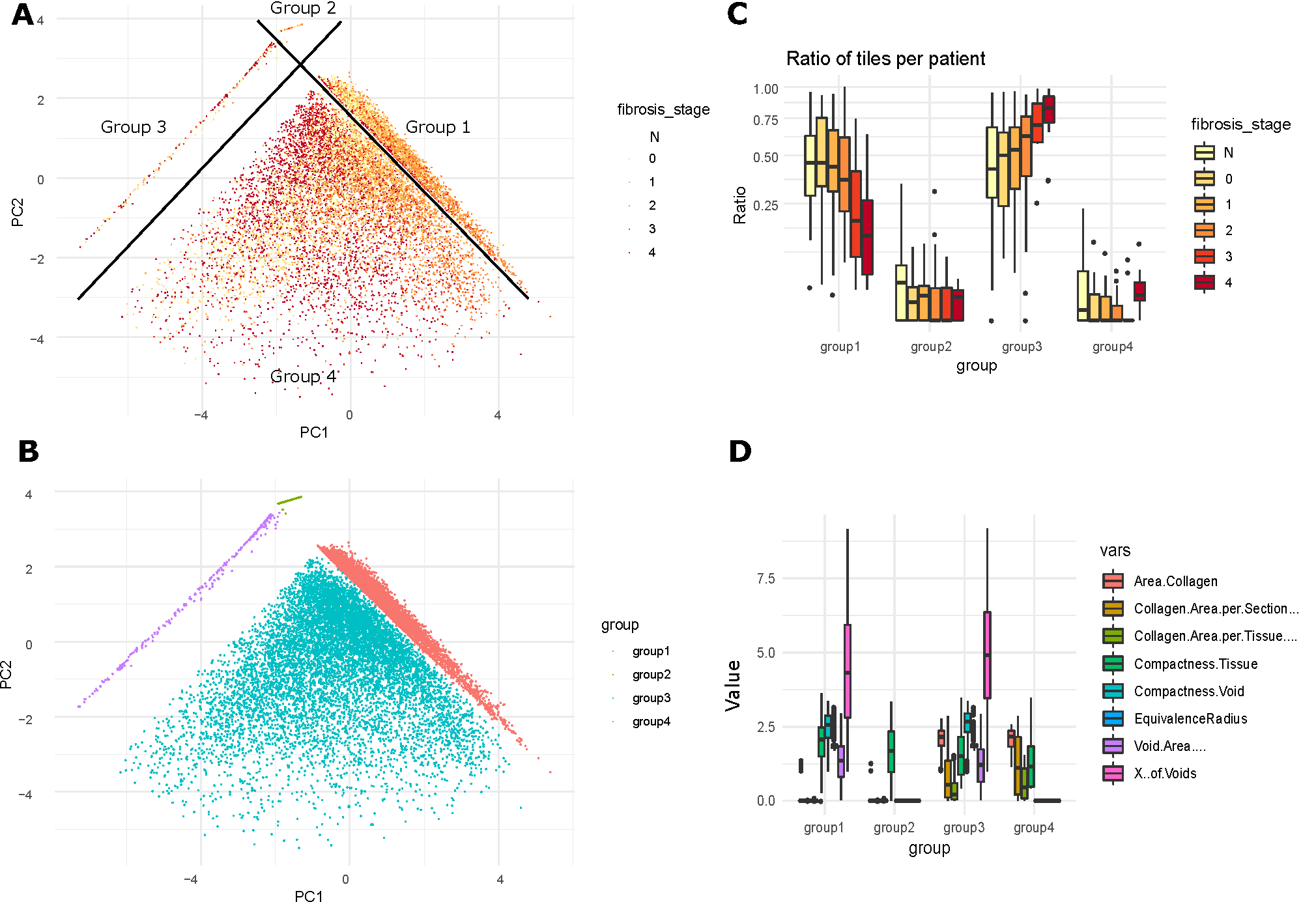


**Fig. S2. Image analysis to derive continuous fibrosis scores.** (A) PCA decomposition of the image tiles colored by fibrosis stage (A) and tile group (B). The tiles appear to cluster in four different groups that correlate strongly with abundance of voids and collagen per tile which again correlate with fibrosis stage .(C) Boxplot with the tile fibrosis stage for each group (D) Boxplot with tile feature distribution for each group.


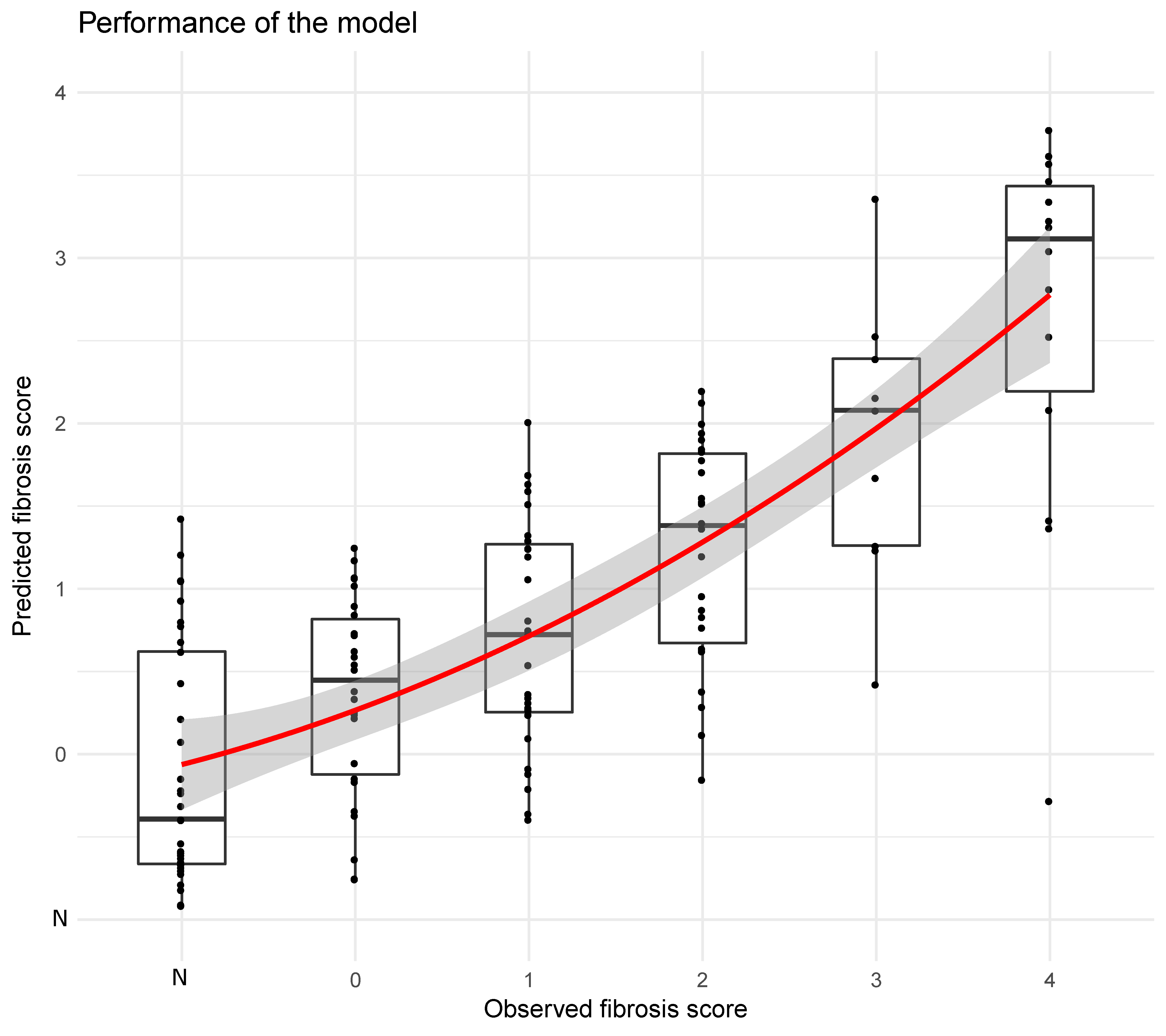


**Fig. S3. Continuous fibrosis score (ImageScore) derived from image features using a non-linear regression model.** The plot depicts the distribution of median predictions for 100 random subsampling rounds on the vertical axis and the original annotations from the pathologists on the horizontal axis.


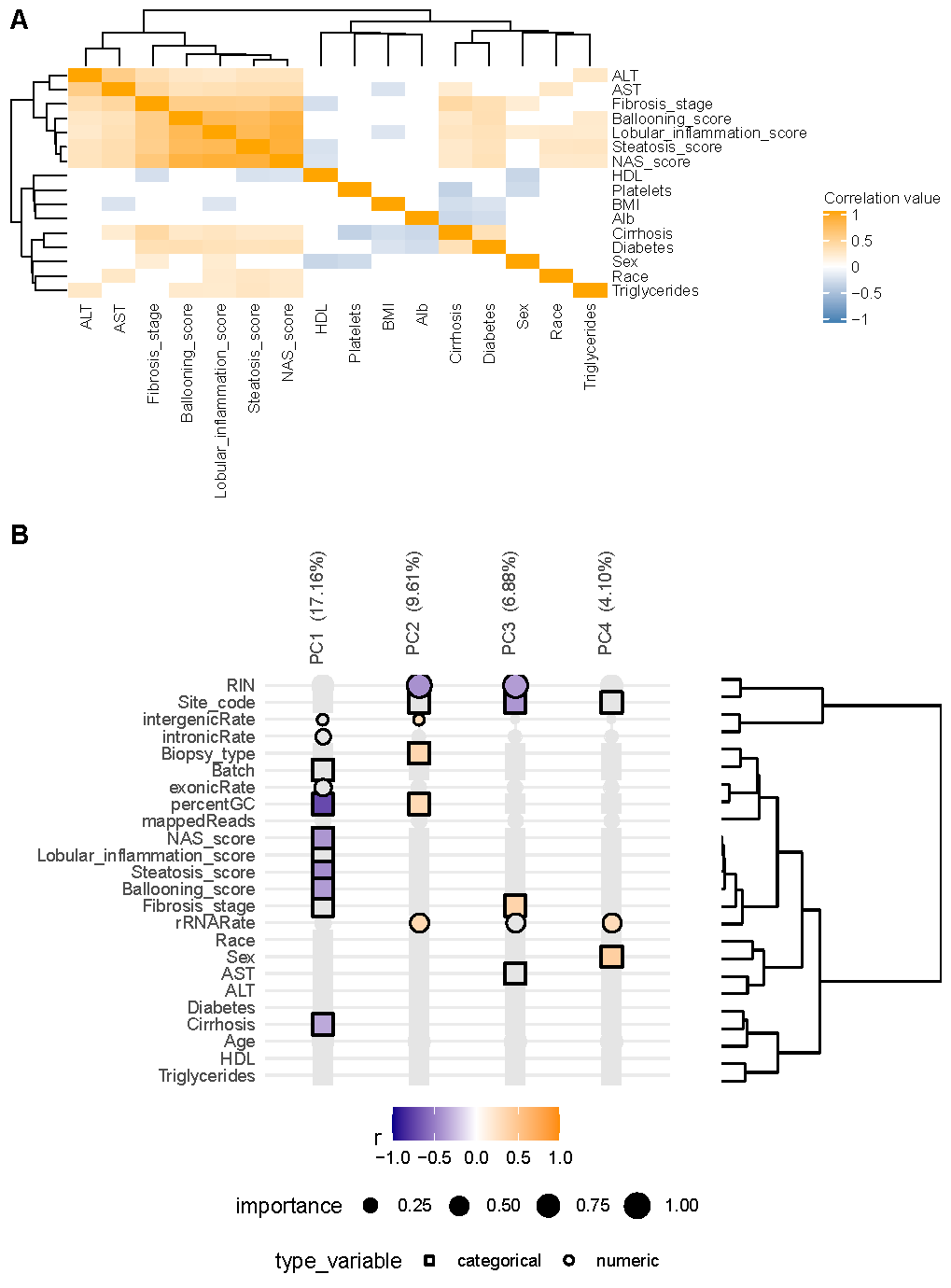


**Fig. S4. (A) Correlation between clinical covariates.** (B) Correlation between first four principal components (PCs) from RNA-Seq analysis with clinical variables and potential confounding factors / covariates.


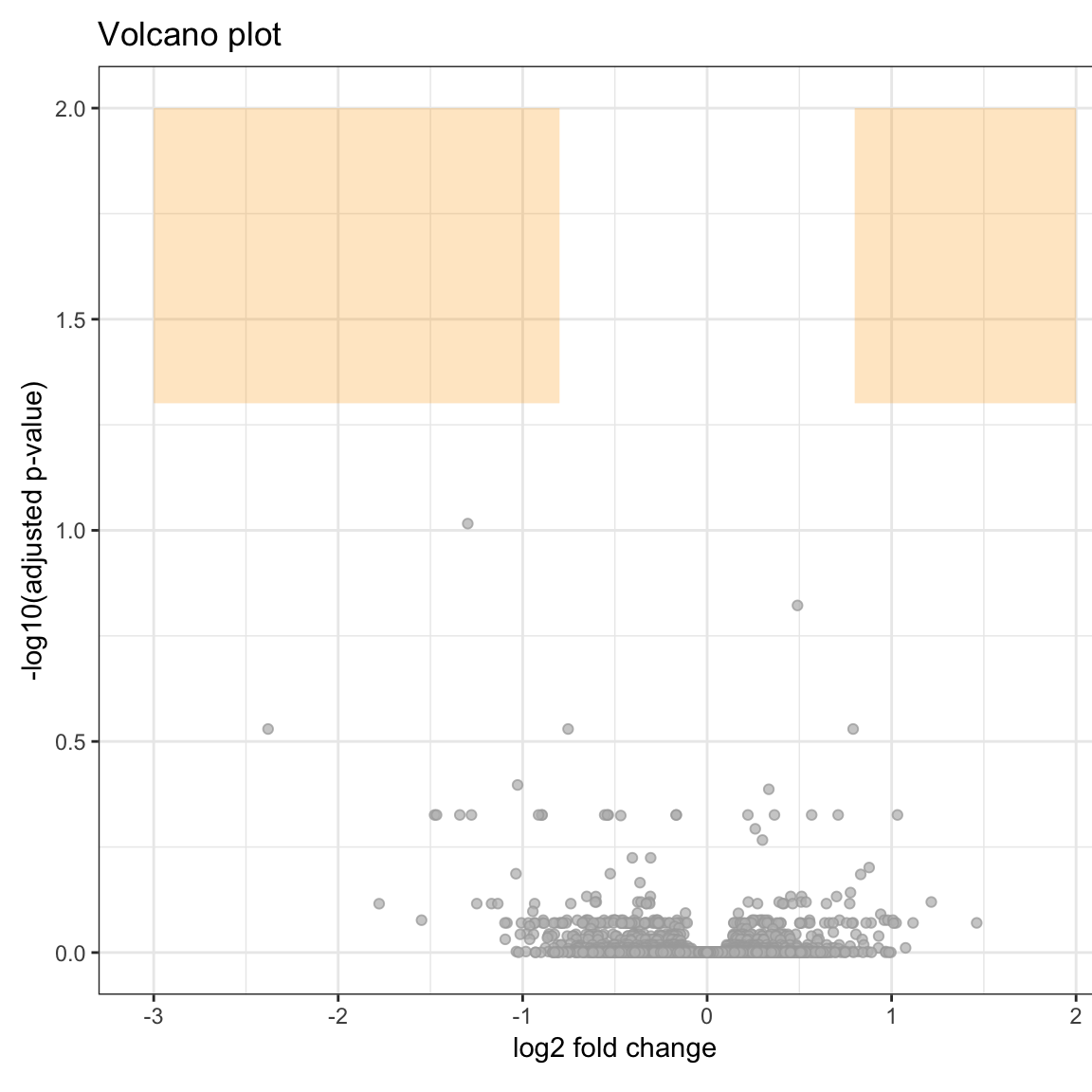

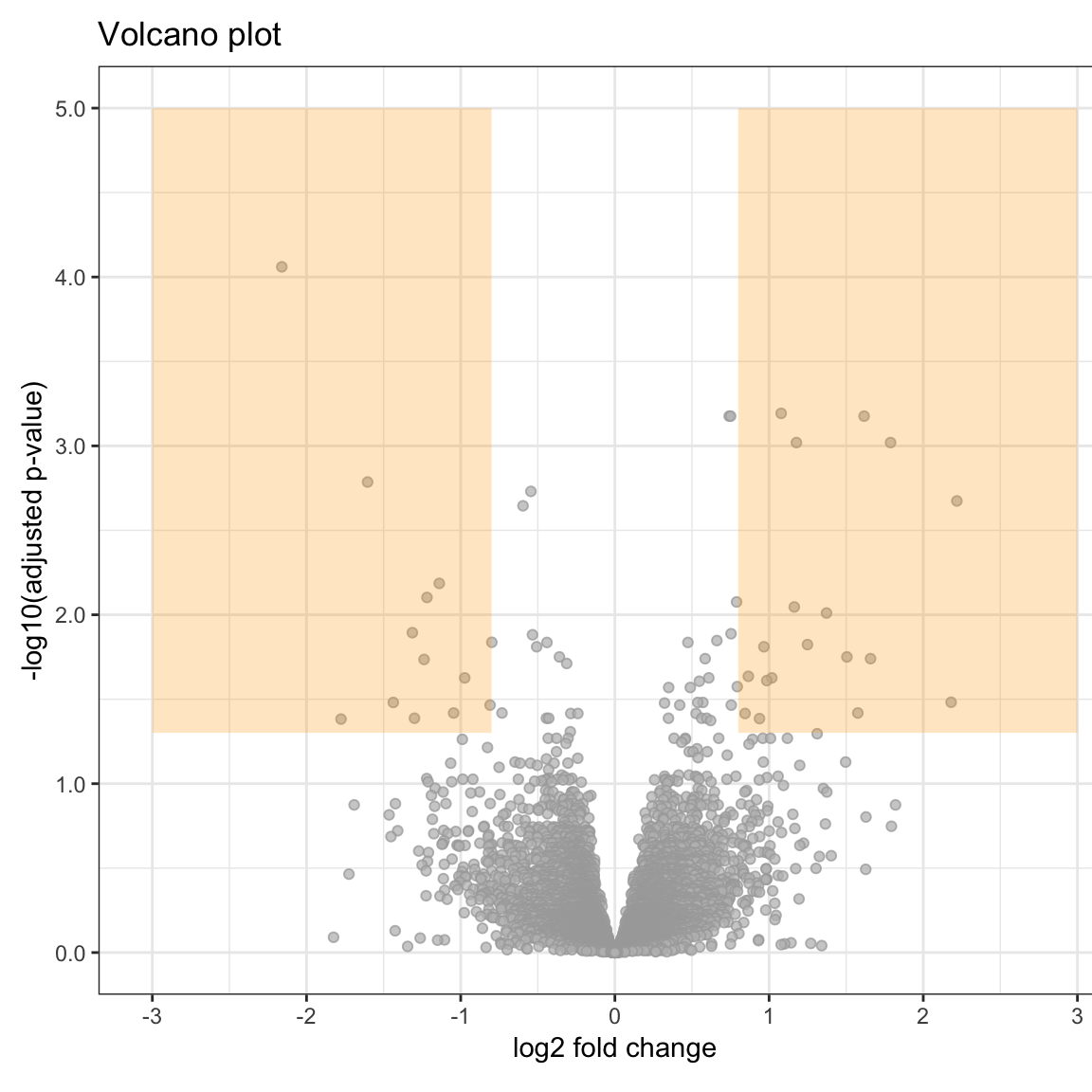


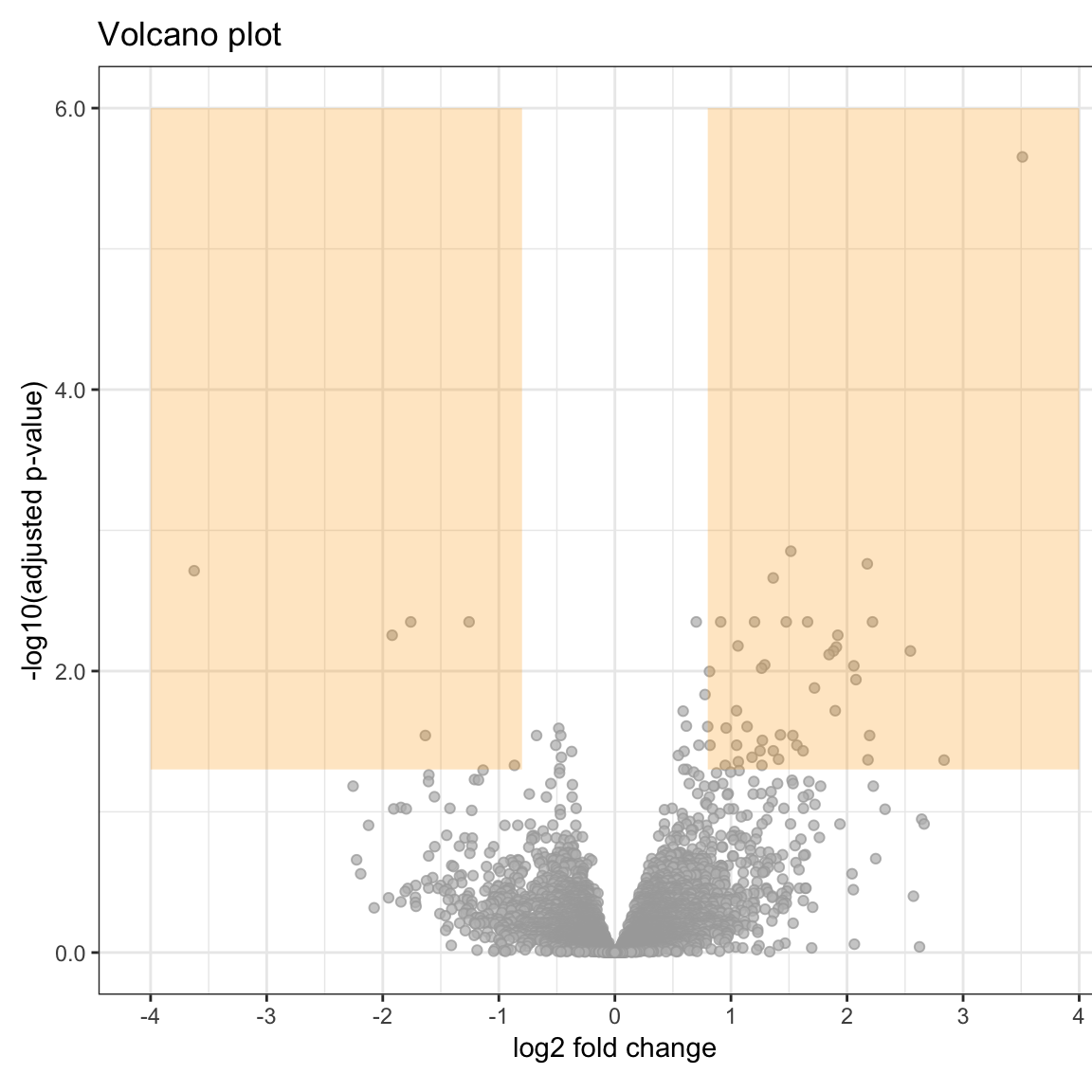

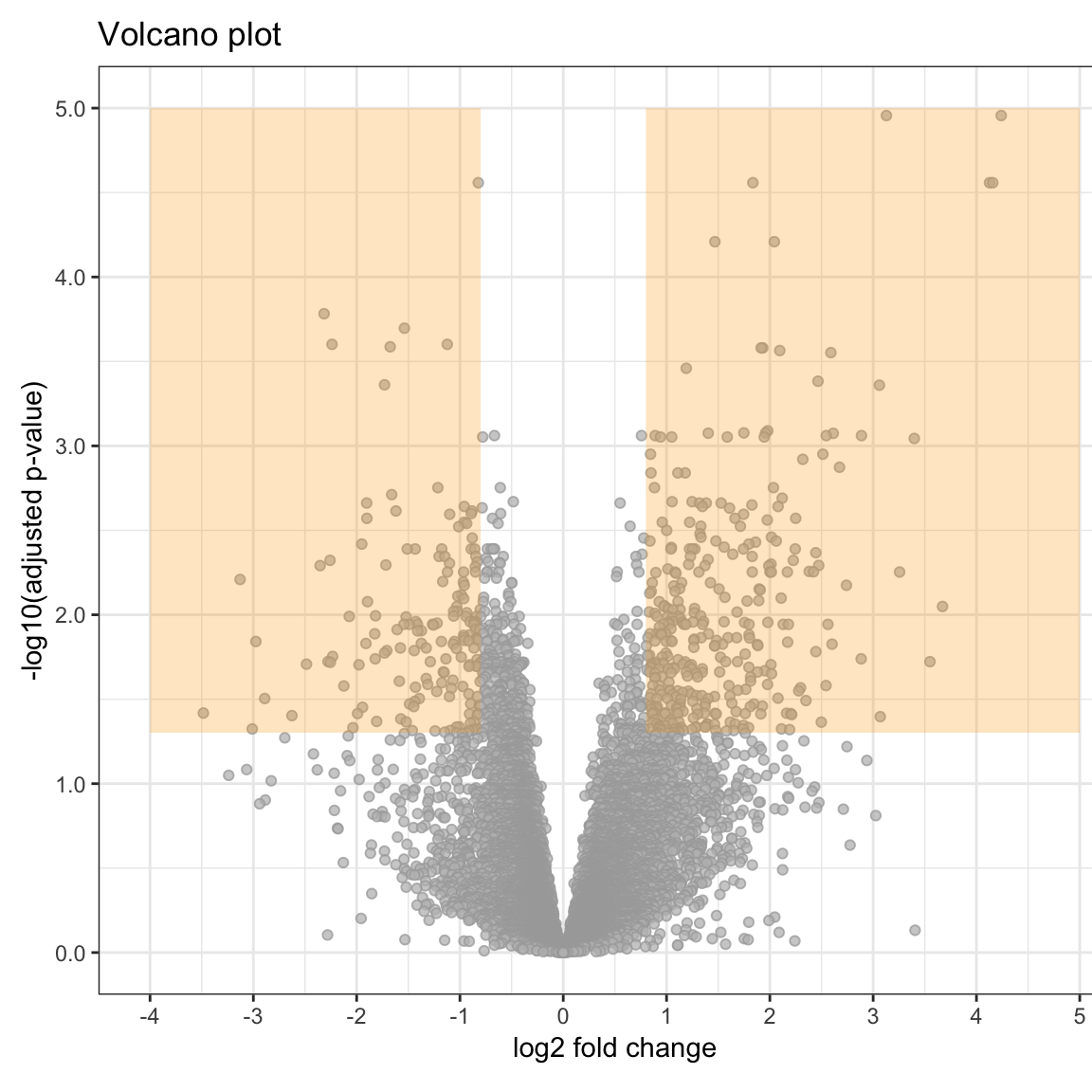


**Fig. S5. Volcano plots of differential gene expression vs. fibrosis grade F0.** The figure shows differential gene expression across fibrosis stages with F1 vs. F0 (upper left), F2 vs. F0 (upper right), F3 vs. F0 (lower left) and F4 vs. F0 (lower right). Yellow shaded background indicates the area of differentially expressed genes according to significance levels padj≤0.05 and |log2 foldchange|≥0.8.

**
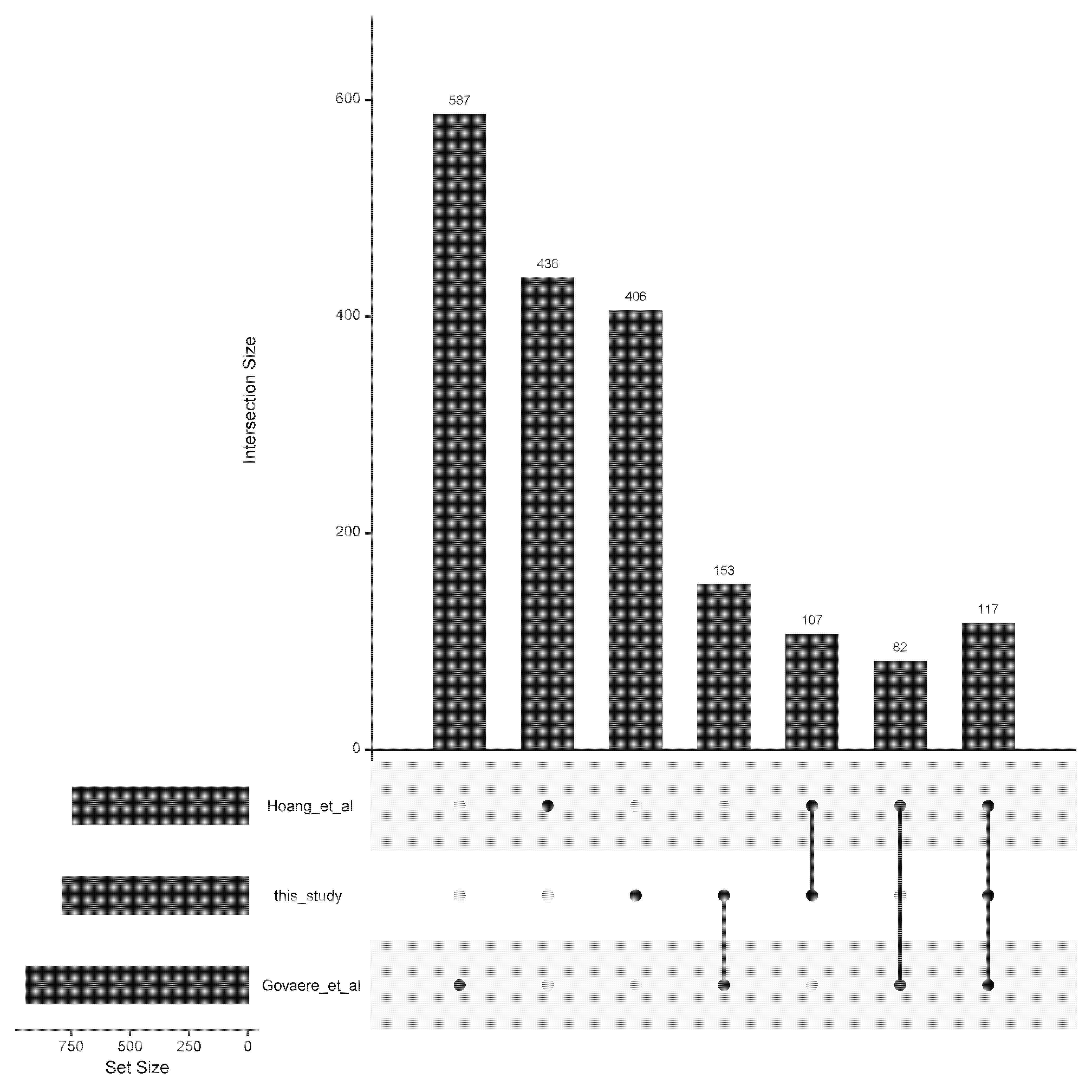
**

**Fig. S6. Gene sets up-regulated with fibrosis in NASH.**  The up-set plot shows the intersections of up-regulated genes observed in the present study with previously published RNASeq studies (see Table S1).

**
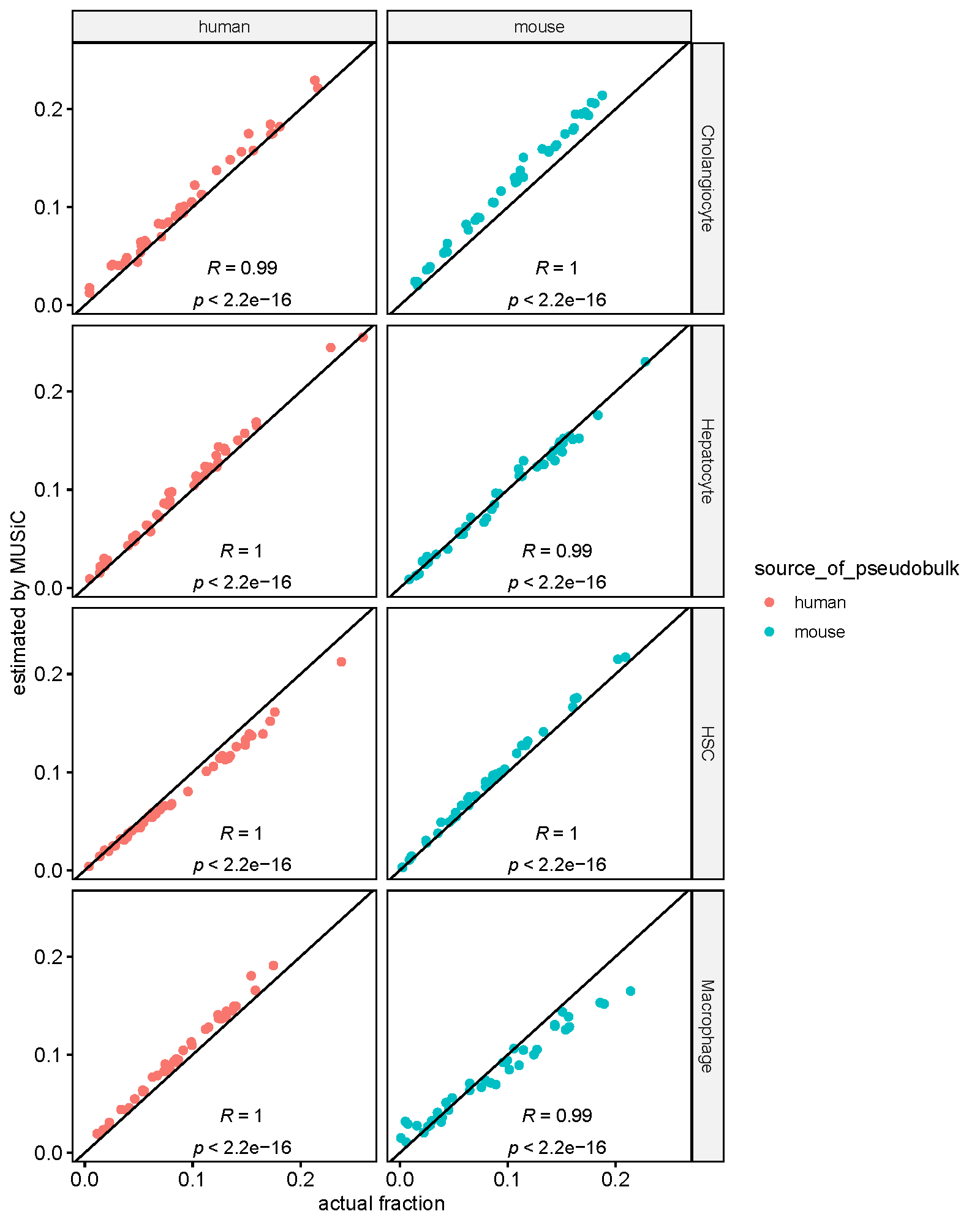
**

**Fig. S7. Validation of cell type deconvolution method with MuSIC using pseudo-bulk data generated from two single cell data sets.** In total, 80 pseudo-bulk samples were simulated from scRNAseq datasets. These scRNAseq datasets comprise of cells from human and mice livers, and 40 pseudo-bulk samples were simulated from each of these cell groups.

**
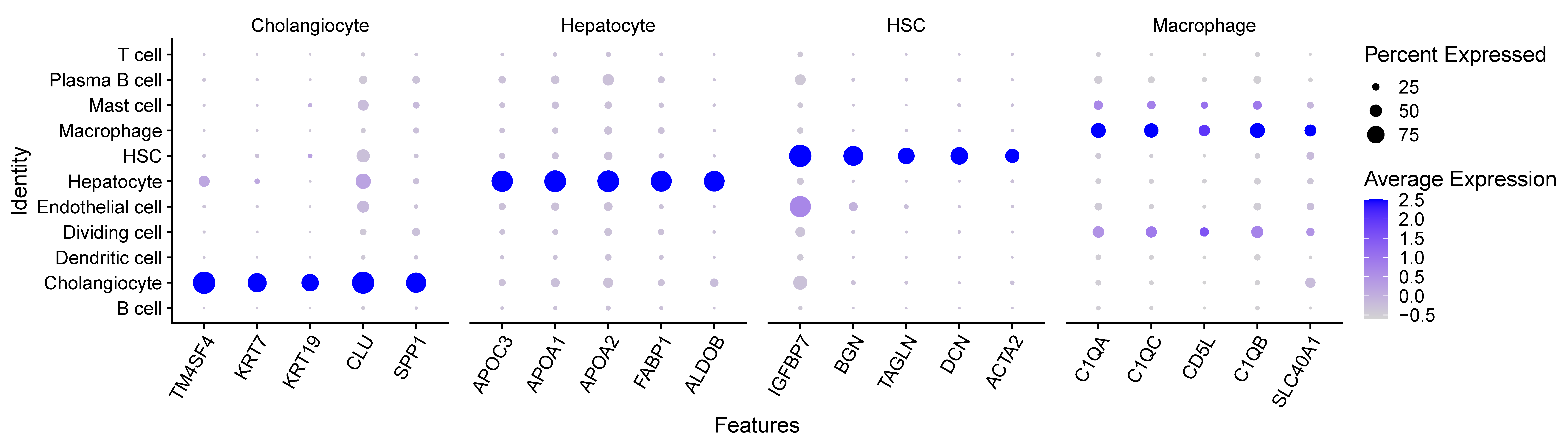
**

**Fig. S8A. Validation of cell type annotation of the combined single cell reference using multiple cell type specific markers for major cell types.** The dotplot shows the abundance of a marker and percentage of cells expressing a marker across cell types (y-axis) for top five markers of the four main cell types (Markers have been derived by findMarker function in Seurat package).

**
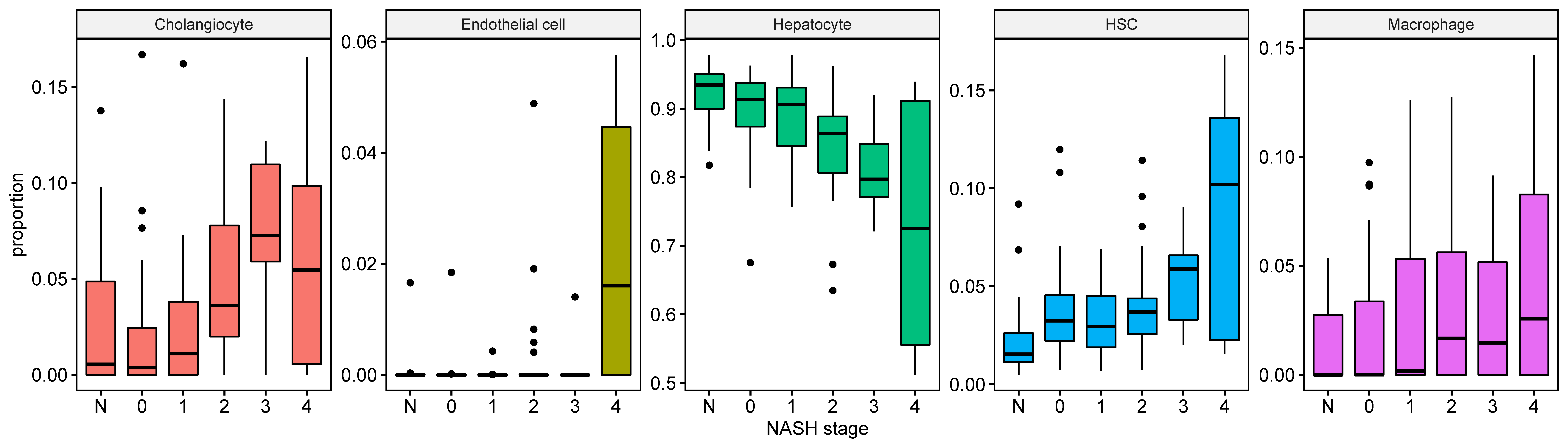
**

**Fig. S8B. Predicted change of cell type proportions across observed NASH fibrosis stage including Endothelial cells.**


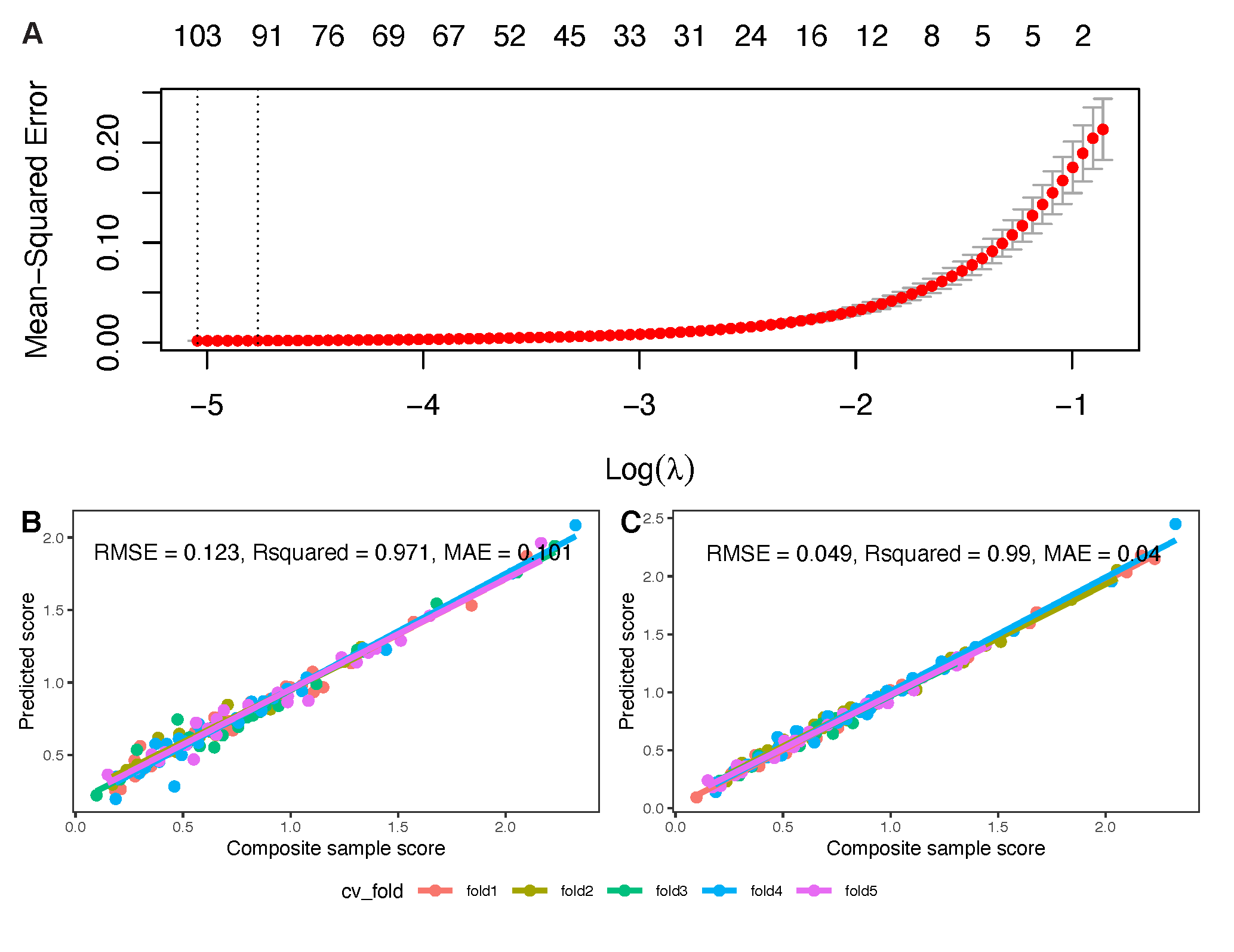


**Fig S9. A. The relationship between log(lambda), Mean-Squared Error and the number of non-zero coefficients (top) in LASSO cross-validation**. (B, C) The results of 5-fold cross-validation for the lambdas that result in an acceptably low and minimum Mean- Squared error.
