# Supplementary material for: Molecular characterization and cell type composition deconvolution of fibrosis in NAFLD
Source: Sci Rep. 2021 Sep 10;11:18045. doi: 10.1038/s41598-021-96966-5 (PMC8433177; doi:10.1038/s41598-021-96966-5)
Supplement: Supplementary file 2 — Supplementary Information 2. [file 41598_2021_96966_MOESM2_ESM.docx]

# Supplemental Table S1

Overview on transcriptomic studies which report genes that are associated with fibrosis in NASH vs. NAFL.

| **Study** | **Method** | **Description** | **Gene set Size** |
| --- | --- | --- | --- |
| Govaere_et_al | RNASeq | Up-regulated in F4 (n=14) vs. baseline F0/F1 (n=85)P^1^ | 939 |
| Hoang_et_al | RNASeq | Positively correlated in with fibrosis stage (ordinal regression, log2ratio ≥ 0.2, FDR≤1%, F0 n=25, F1 n=28, F2 n=9, F3 n=14, F4n=2)P^2^ | 742 |
| Lefebvre_et_al | Microarray | Gene differentially expressed in NAFLD Fibrosis vs. no fibrosis (F0, n=47 vs. F2-4, n=13)P^3^ | 86 |
| Moylan_et_al | Microarray | Top differentially expressed genes in “severe” NAFLD (fibrosis ≥ F3, n=32) vs. “mild” NAFLD (fibrosis ≤ F1, n=40)P^4^ | 98 |
| Suppli_et_al | RNASeq | Differentially expressed gene in NASH (hepatocyte ballooning and early fibrosis, F1 n=12/16, F2 1/16) vs. NAFL (no ballooning, F0 n=14/15, F1 n=1/15; FDR ≤5%, log2ratio ≥0.28)P^5^ | 112 |
| This_study | RNASeq | Genes positively correlated with fibrosis stage (see cluster #2 and #3 in Fig. 1) | 783 |

1. Govaere, O. *et al.* Transcriptomic profiling across the nonalcoholic fatty liver disease spectrum reveals gene signatures for steatohepatitis and fibrosis. *Sci Transl Med* **12**, eaba4448 (2020).

2. Hoang, S. A. *et al.* Gene Expression Predicts Histological Severity and Reveals Distinct Molecular Profiles of Nonalcoholic Fatty Liver Disease. *Sci Rep-uk* **9**, 1–14 (2019).

3. Lefebvre, P. *et al.* Interspecies NASH disease activity whole-genome profiling identifies a fibrogenic role of PPARalpha-regulated dermatopontin. *Jci Insight* **2**, e92264 (2017).

4. Moylan, C. A. *et al.* Hepatic gene expression profiles differentiate presymptomatic patients with mild versus severe nonalcoholic fatty liver disease. *Hepatology* **59**, 471–482 (2014).

5. Suppli, M. P. *et al.* Hepatic transcriptome signatures in patients with varying degrees of nonalcoholic fatty liver disease compared with healthy normal-weight individuals. *Am J Physiol-gastr L* **316**, G462–G472 (2019).

# Supplemental Table S2

Most robustly enriched biological pathways from WikiPathways (WP), the comprehensive resource of mammalian protein complexes (CORUM), Gene Ontology (GO) and Reactome (REAC) in the 98-gene signature.

| Source | term_name | | term_id | adjusted_p_value | Signature genes | | | |
| --- | --- | --- | --- | --- | --- | --- | --- | --- |
| WP | miRNA targets in ECM and membrane receptors | | WP:WP2911 | 0.000284 | *COL5A1,COL4A2,THBS2,COL4A1* | | | |
| CORUM | pERK-vimentin-KPNA2 complex | | CORUM:6509 | 0.002485 | *VIM,KPNA2* | | | |
| GO:CC | basement membrane | | GO:0005604 | 0.009495 | *LAMC3,COL5A1,COL4A2,THBS2,COL4A1* | | | |
| GO:CC | complex of collagen trimers | | GO:0098644 | 0.009495 | *COL5A1,COL4A2,COL4A1* | | | |
| REAC | Non-integrin membrane-ECM interactions | | REAC:R-HSA-3000171 | 0.012618 | *LAMC3,COL5A1,COL4A2,COL4A1* | | | |
| REAC | Signaling by PDGF | | REAC:R-HSA-186797 | 0.012618 | *COL5A1,COL4A2,THBS2,COL4A1* | | | |
| GO:CC | extracellular matrix | | GO:0031012 | 0.01322 | *LAMC3,PKM,COL5A1,COL4A2,SERPINB9,THBS2,COL4A1,S100A4,ADAMTSL2* | | | |
| GO:CC | collagen-containing extracellular matrix | | GO:0062023 | 0.01322 | *LAMC3,PKM,COL5A1,COL4A2,SERPINB9,THBS2,COL4A1,S100A4* | | | |
| REAC | ECM proteoglycans | | REAC:R-HSA-3000178 | 0.017148 | *COL5A1,COL4A2,ITGAX,COL4A1* | | | |
| REAC | Laminin interactions | | REAC:R-HSA-3000157 | 0.017148 | *LAMC3,COL4A2,COL4A1* | | | |
| REAC | Integrin cell surface interactions | | REAC:R-HSA-216083 | 0.021181 | *COL5A1,COL4A2,ITGAX,COL4A1* | | | |
| WP | Focal Adhesion-PI3K-Akt-mTOR-signaling pathway | | WP:WP3932 | 0.02145 | *LAMC3,PIK3IP1,COL5A1,COL4A2,ITGAX,THBS2,COL4A1* | | | |
| CORUM | ITGA4-ITGB1-THBS2 complex | | CORUM:2428 | 0.025154 | *THBS2* | | | |
| CORUM | ING4 complex (ING4, JADE1, KAT7, MEAF6) | | CORUM:1352 | 0.025154 | *MEAF6* | | | |
| CORUM | ZAP70-CRKL-WIPF1-WAS complex | | CORUM:2510 | 0.025154 | *WIPF1* | | | |
| CORUM | Alpha-dystrobrevin-ZO-1-actin complex | | CORUM:1041 | 0.025154 | *DTNA* | | | |
| GO:MF | glycosaminoglycan binding | | GO:0005539 | 0.043833 | *F11,CXCL6,COL5A1,GPNMB,HK1,THBS2* | | | |
| GO:MF | extracellular matrix structural constituent conferring tensile strength | | GO:0030020 | 0.043833 | *COL5A1,COL4A2,COL4A1* | | | |
| GO:MF | amino acid transmembrane transporter activity | | GO:0015171 | 0.043833 | *SLC1A3,SLC16A10,SLC1A7,SLC38A11* | | | |
| GO:MF | heparin binding | | GO:0008201 | 0.043833 | *F11,CXCL6,COL5A1,GPNMB,THBS2* | | | |
| WP | Computational Model of Aerobic Glycolysis | | WP:WP4629 | 0.045708 | *PKM,HK1* | | | |
| KEGG | Amoebiasis | | KEGG:05146 | 0.046223 | *LAMC3,COL4A2,SERPINB9,COL4A1* | | | |
| KEGG | Protein digestion and absorption | | KEGG:04974 | 0.046223 | *SLC16A10,COL5A1,COL4A2,COL4A1* | | | |
| KEGG | ECM-receptor interaction | | KEGG:04512 | 0.046223 | *LAMC3,COL4A2,THBS2,COL4A1* | | | |
|  | |  | | | |  |  |  |
