# Supplementary material for: Molecular characterization and cell type composition deconvolution of fibrosis in NAFLD
Source: Sci Rep. 2021 Sep 10;11:18045. doi: 10.1038/s41598-021-96966-5 (PMC8433177; doi:10.1038/s41598-021-96966-5)
Supplement: Supplementary file 4 — Supplementary Information 4. [file 41598_2021_96966_MOESM4_ESM.docx]

# Supplemental Methods

### Validation of the cell type deconvolution model

We validated the deconvolution model to predict liver cell type proportions using pseudo-bulk mixture samples which have been generated from the liver single cell reference data sets. Accordingly, we simulated 40 pseudo-bulk samples from human scRNA-Seq reference^1^ and 40 pseudo-bulk samples from mouse scRNA-Seq reference^2^, respectively. In each of the simulated pseudo-bulk sample, the number of resampled cells for each cell type is derived from a multinomial distribution:

$x_{ij} \sim M\left( N, p_{1j,} p_{2j, \ldots,}p_{nj,} \right)$ **Eq. (1)**

M denoting multinomial distribution with parameters N = 8000 and event probabilities

$p_{ij}, \sum_{i=1}^{n} p_{ij}=1$ **Eq. (2)**

where i stands for the index of cell type and j stands for the index of pseudo-bulk sample. The event probabilities (cell type fractions), which should add up to 1, are derived from

$p_{ij}= \frac{w_{ij}}{\sum_{i=1}^{n} w_{ij}}, where w_{ij} \sim unif\left( 0,1 \right)$ **Eq. (3)**

Whereby w_ij_ corresponds to a random variable taken from the uniform distribution.

For deconvolution of our bulk data, we first computed transcripts per million (TPM) values for all samples and then estimated the proportion of each cell type in each sample. We observed robust and consistent cell type proportions because the results obtained for male vs. female samples were highly correlated (r>0.9, P value < 1e-16), and because the results were very similar to the output from CIBERSORTx ^3^, a second deconvolution method which allows for explicit batch correction.

### Combined single cell reference data

Comprehensive and relevant liver scRNA-Seq datasets from human ^1^ and mice ^2^ and the Harmony method ^4^ were used to derive an integrated, batch corrected, and aligned reference dataset for cell type deconvolution. Accordingly, raw data were pre-processed with STARsolo (v2.7.3a) ^5^ to generate the gene count matrix. Using Seurat ^6^, we removed cells with <300 or >6000 genes detected, and with >15% of UMIs from mitochondrial RNAs. For the remaining cells, we normalized the raw read counts to log-transformed per 10k UMIs. We selected the top 2000 most variable genes for PCA after scaling. The top 40 principal components (PCs) were used for graph-based clustering with resolution 1.2 by shared nearest neighbor (SNN). To annotate the cell types, we referred to markers in the original publications for each dataset and manually annotated cell clusters. To obtain an integrated reference, we used “one-to-one" ortholog mappings of mouse and human gene IDs annotated in the ENSEMBL database. We then merged the two datasets into a joint gene count matrix with shared orthologs and aligned them with Harmony ^4^ by removing batch effects of cell type enrichment and disease status or treatment group. We re-clustered the cells in the aligned data and re-assigned cell types. We harmonized the cell type nomenclature from the two datasets by primarily applying the nomenclature from the mouse study to rename the cell types in the human study i.e., mesenchyme to HSCs. Finally, we excluded species-specific cell clusters from the integrated scRNA-Seq dataset for further analysis.

# Supplemental References

1 Ramachandran, P. *et al.* Resolving the fibrotic niche of human liver cirrhosis at single-cell level. *Nature* **575**, 512-518, doi:10.1038/s41586-019-1631-3 PMID - 31597160 (2019).

2 Xiong, X. *et al.* Landscape of Intercellular Crosstalk in Healthy and NASH Liver Revealed by Single-Cell Secretome Gene Analysis. *Mol. Cell* **75**, 644-660.e645, doi:10.1016/j.molcel.2019.07.028 (2019).

3 Newman, A. M. *et al.* Determining cell type abundance and expression from bulk tissues with digital cytometry. *Nature Biotechnology* **37**, 773-782, doi:10.1038/s41587-019-0114-2 (2019).

4 Korsunsky, I. *et al.* Fast, sensitive and accurate integration of single-cell data with Harmony. *Nature Methods* **16**, 1289-1296, doi:10.1038/s41592-019-0619-0 (2019).

5 Blibaum, A., Werner, J. & Dobin, A. STARsolo: single-cell RNA-seq analyses beyond gene expression. *F1000Research* **8** (2019).

6 Stuart, T. *et al.* Comprehensive integration of single-cell data. *Cell* **177**, 1888-1902. e1821 (2019).
